# *In vitro* identification of GABA-producing psychobiotic candidates from human breast milk

**DOI:** 10.3389/fmicb.2026.1715064

**Published:** 2026-04-15

**Authors:** Murali Anagha, Roshni Ramachandran, Gangaraju Divyashri, Sindhu O., Somashekar A. R., M. V. Krithika, T. P. Krishna Murthy

**Affiliations:** 1 Department of Biotechnology, M. S. Ramaiah Institute of Technology, Bangalore, Karnataka, India; 2 Head, Bioproducts Research, Iom Bioworks Pvt. Ltd., Bangalore, Karnataka, India; 3 Department of Pediatrics, Ramaiah Medical College, Bangalore, Karnataka, India

**Keywords:** breastmilk, GABA, gut-brain axis, mental health, neuropsychiatry, probiotics, psychobiotics

## Abstract

Human breastmilk is a rich source of beneficial microbiota and bioactive compounds, yet its potential as a reservoir of psychobiotics remains underexplored. In this study, breastmilk samples were aseptically collected from healthy lactating mothers and cultured using MRS media. A total of 188 bacterial isolates were screened for gamma-aminobutyric acid (GABA) production using monosodium glutamate (MSG) as a substrate, with quantification by High-Performance Liquid Chromatography (HPLC). Several isolates, mainly Gram-positive cocci of the *Enterococcus* genus, showed GABA production ranging from 0.3343 to 0.8471 g/L. The most potent isolate identified as *Enterococcus faecalis* RIT BM_S15-3_ through 16S rRNA sequencing, exhibited notable resistance to gastrointestinal conditions and antimicrobial activity. Further, fermentative GABA production was optimized by adjusting pH, temperature, and MSG concentration. These findings highlight breastmilk as a promising source of GABA-producing bacteria with potential psychobiotic relevance. However, comprehensive strain-specific safety and *in vivo* evaluations are required before considering applications in functional foods or microbiota-based strategies targeting the gut–brain axis.

## Graphical abstract

**Figure:**
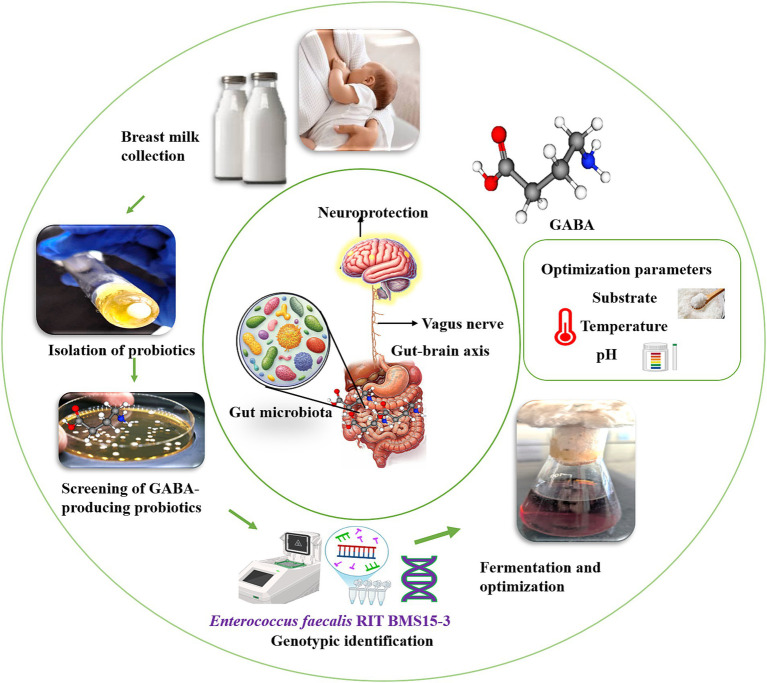

## 1 Introduction

Worldwide, there is a rise in the prevalence of mental health disorders such as depression, anxiety, and stress. According to WHO, it is a significant global concern, comprising 13% of the world’s population experiencing mental illness, that is 970 million affected people (Liu et al., 2025). These conditions have initiated a prominent shift toward preventive approaches for mental well-being, with an increased focus on the gut-brain axis as an etiology. Conventional pharmacotherapy for managing mood disorder consist of selective serotonin reuptake inhibitors (SSRIs), benzodiazepines, and GABA analogs like gabapentin and pregabalin (Ibrahim et al., 2022; Patsalos, 2022; Nwankwo et al., 2024) while often effective, are frequently associated with adverse effects ranging from sedation and cognitive impairment to gastrointestinal distress, weight gain, dependency, and more severe complications such as myoclonus, ataxia, or suicidal ideation, particularly with long-term use or in sensitive populations (Christodoulou, 2020; Elias and Rosenblum, 2024). Given these restrictions, the neuroscience and psychobiology community has moved toward studying the gut-brain axis. This complex two-way communication system connects the central nervous system (CNS) with the enteric nervous system and is heavily impacted by the gut microbiota (Bakshi et al., 2024). Recent research in neurogastroenterology, microbiome science, and neuropsychology has started to reveal how the various microbial communities living in the digestive system can influence important neurological functions through processes related to metabolism, immunity, hormones, and nerve pathways (Lopez et al., 2025; Sun et al., 2025). The concept of microorganisms that affect mood and behavior is not speculative anymore but supported by rising evidence, involving findings from potential preclinical models and human trials demonstrating probiotic effect on anxiety, depressive symptoms, cognitive flexibility, and neuroendocrine responses to stress (Vitellio et al., 2020). Central to this emerging field is the identification of a special class of probiotics known as psychobiotics.

According to FAO/WHO, probiotics are live microorganisms that when administered in adequate amounts, benefits the host health. Psychobiotics are probiotics, primarily bacteria, which, when ingested in sufficient amounts, confer mental health benefits (Oleskin and Shenderov, 2019; Kapreliants and Zhuk, 2021; Kyei-Baffour et al., 2025). Among the most critical contributions of psychobiotics is their ability to synthesize neuroactive molecules such as gamma-aminobutyric acid (GABA), serotonin (5-HT), dopamine, acetylcholine, and brain-derived neurotrophic factor (BDNF), each of which plays an essential role in neurotransmission (Imran et al., 2025), mood regulation (Shabel et al., 2014), circadian rhythm control (Meyers and Tamir, 2024), anxiety reduction (Hagan et al., 2022), and cognitive processing (Braga et al., 2024). Our focus of interest is GABA, which is the major inhibitory neurotransmitter in the human brain and spinal cord, functioning to reduce neuronal excitability and promote a sense of calm, balance, and resilience to stress (Shabel et al., 2014). GABA controls nearly 40% of inhibitory synapses in adult vertebrates (Singh and Zhawar, 2024). Historically, synthetic GABA analogs have been used to manipulate GABAergic pathways (Heath et al., 2023; Lima-Silveira et al., 2022; Munoz et al., 2025; Liu et al., 2022; Somekawa et al., 2024; Mick and McCormick, 2024; Ferreira et al., 2022; Rashid et al., 2024), yet these are often not well tolerated and may lead to undesirable drug–drug interactions. For decades, medications with GABA were frequently used to treat various mental and neurological conditions like generalized anxiety, insomnia, major depression, schizophrenia, and epilepsy (de la Ochoa- Paz et al., 2021). Hence, the therapeutic potential of natural, microbial-derived GABA is being increasingly recognized for its biocompatibility, functional versatility, and potential for integration into functional foods and dietary supplements (Kim et al., 2025). The presence of probiotics in the gut that naturally generate GABA can activate GABA signaling through the gut-brain axis, influencing both brain activity and behavior (Cui et al., 2025). This finding opens up new possibilities for enhancing mental health support and creating interventions (Denysov et al., 2023; Oyovwi and Udi, 2025).

Certain strains of lactic acid bacteria (LAB) and *Bifidobacterium*, such as *L. plantarum*, *L. brevis*, *L. buchneri*, *L. rhamnosus*, *Lactococcus lactis*, and *E. faecium*, have been identified for their ability to produce GABA *via* the enzymatic decarboxylation of glutamate catalyzed by glutamate decarboxylase (GAD), a reaction that also consumes intracellular protons and helps bacteria survive in acidic environments, thus conferring dual benefits in food fermentation and probiotic survival (Cho et al., 2007; Takagi et al., 2022; Ayyash and Liu, 2023). Recent studies explored the state-of-the-art findings in strain development strategies for industrial LAB (Yehuala et al., 2025), *Corynebacterium glutamicum* (Yao et al., 2023), and *Escherichia coli* MTCC 42 (Reiter et al., 2024) to augment bioproduction of GABA (Luo et al., 2021). Probiotics such as *L. buchneri* from kimchi (Lee and Kim, 2023), *Lactococcus lactis subsp. Lactis* (Monteiro et al., 2024), *L. brevis* (Bock et al., 2023), *L. plantarum* (Choi et al., 2022), probiotic NCL912 (Casertano et al., 2024), and *E. faecium* NCIM 5593 (Gangaraju et al., 2014) have already been studied to enhance the production of GABA. These findings have opened up the possibility of formulating next-generation probiotics targeted at improving mental health through gut microbial modulation.

Despite this promising landscape, a major gap exists in exploring novel, naturally safe, and personalized sources of psychobiotics that are tailored for early life and sensitive populations. One such untapped reservoir is human breastmilk—a dynamic, bioactive fluid that not only provides essential nutrition to infants but also delivers immunological protection, hormonal signals, and diverse microbial species that influence the neonatal gut microbiome, immune system maturation, and brain development (Panigrahi, 2024; Ghith et al., 2025). Breastmilk is known to contain beneficial strains such as *L. reuteri*, *L. fermentum*, and *B. longum,* which play vital roles in maintaining gut homeostasis and preventing colonization by pathogens in neonates (Faizullina and Ukraintsev, 2024). Maternal genetics, health, delivery method, and dietary habits affect these microbial groups, providing a naturally tailored microbial signature that could be ideal for probiotic use. Furthermore, breastmilk comprises bioactive components such as human milk oligosaccharides (HMOs), secretory IgA, lactoferrin, and cytokines that aid the functionality and survival of these microbes (Karim, 2023; Greenwood et al., 2024). Given its generally recognized as safe (GRAS) status, biocompatibility, and immunological significance, human breastmilk presents a unique opportunity to isolate novel GABA-producing probiotics for potential application in mental health support across age groups. It not only provides insights into their major role in mental health promotion, but also in functional food applications. A part of this investigation was to examine the reaction parameters (Alang et al., 2020), such as temperature, substrate concentration, and pH, and how these affect the production of GABA. The current work aims to isolate, screen, characterize, and evaluate human breast milk–derived psychobiotics, identify the top GABA-producing isolate, and assess reaction parameters influencing GABA production (Lu et al., 2009).

## 2 Materials and methods

### 2.1 Chemicals and reagents

De Man Rogosa and Sharpe (MRS) broth granulated, MRS agar, Monosodium glutamate (MSG), Nutrient broth, Muller Hinton (MH) broth and MH agar were procured from Hi Media, India. Reagents of HiIMViC™ Biochemical Test Kit (KB001) from Hi Media, Mumbai, were used for biochemical tests. Carbohydrates from HiMedia, Mumbai (K024-1KT) were used for carbohydrate utilization tests. Gram staining kit (Hi-Media K001-1KT) was obtained from Mumbai, India. Sheep blood agar was obtained from Tern Biotech, Puducherry. Alpha-amylase, Lipase and trypsin were obtained from SRL India. All other chemicals and reagents used were of analytical grade.

### 2.2 Microorganisms

GABA-producing bacteria were isolated from human breast milk. A total of 10–15 mL of breast milk was aseptically collected from each of 17 healthy lactating mothers of newborn infants at Ramaiah Medical College Hospital. Before sample collection, the nipple area was disinfected with ethanol to minimize contamination, and informed consent was obtained from all participants in accordance with ethical guidelines. Additionally, indicator strains classified as Gram-positive including (*Staphylococcus aureus* MTCC 96, *Bacillus cereus* MTCC 430) and Gram-negative (*Pseudomonas aeruginosa* MTCC 424, *Escherichia coli* MTCC 42) were procured from the Microbial Type Culture Collection and Gene Bank (MTCC), CSIR–Institute of Microbial Technology, Chandigarh, for use in antimicrobial activity assays.

### 2.3 Human breastmilk sample collection

#### 2.3.1 Selection of subjects

A total of 17 healthy lactating mothers were recruited from the postnatal ward of the Neonatology Department at Ramaiah Medical College Hospital, Bengaluru, and voluntarily participated in the study. Ethical approval for the study was obtained from the Institutional Ethics Committee of Ramaiah Medical College (Certificate No: MSRMC/EC/AP-02/10–2024). Written informed consent, including participant signatures, was secured from all individuals prior to sample collection, in accordance with institutional and ethical guidelines. Clinical and demographic information for each participant was recorded using a structured study proforma. The recruited mothers were between 18 and 35 years of age, with a height range of 145–170 cm and a body weight between 50–75 kg. Inclusion criteria included healthy lactating women with no history of maternal or pre or postnatal complications. Exclusion criteria comprised individuals with known infectious diseases such as HIV or Hepatitis, those who had received antibiotics during pregnancy, and those who had experienced illness within 3 months before sample donation. Random selection was applied to ensure unbiased sample representation.

#### 2.3.2 Collection of human breastmilk

A total of 10–15 mL of breast milk was aseptically collected from 17 healthy lactating women within the first to third week postpartum. To minimize contamination from skin microbiota, the mammary areola was thoroughly cleansed with 70% ethanol, as described by Sumalata (2023). The initial few drops of milk were discarded, and the remaining volume was manually expressed into sterile Falcon tubes. Samples were immediately transported to the laboratory in an ice box and processed promptly upon arrival. Any remaining portions of the samples were stored at −20 °C for further analysis.

### 2.4 Isolation and identification of psychobiotics

The samples collected were subjected to screening of potential probiotics using standard microbiological methods. Briefly, 1 mL of each breast milk sample was serially diluted in 9 mL of sterile phosphate-buffered saline (PBS) to obtain dilutions ranging from 10^−1^ to 10^−5^. Aliquots from these dilutions were streaked in triplicate onto MRS agar plates using the quadrant streak method. Plates were incubated at 37 °C for 24 h, followed by three to four rounds of subculturing to obtain pure and morphologically distinct bacterial colonies. Individual colonies were then inoculated into MRS broth and incubated at 37 °C for 24 h. After incubation, plates were examined for diverse colony morphologies, and representative colonies from each dilution were isolated (Sumalata, 2023). Actively growing cultures were further inoculated at 10% (v/v) into MRS broth supplemented with 3% monosodium glutamate (MSG) and incubated under static conditions at 37 °C for 48 h to induce GABA production. All isolates were preserved as glycerol stocks and cryopreserved at −20 °C for future use. Further, isolated microbial single colonies suspected as probiotics were selected and identified using standard morphological and biochemical methods, then confirmed with the help of molecular methods (Sumalata, 2023).

#### 2.4.1 Morphological identification

The isolated pure cultures were subjected to identification using macroscopic and microscopic appearance to analyze their morphological characteristics like size, shape, margin, color, and texture of the colonies (Holt et al., 1994). Various bacterial colonies were observed under a light microscope with 100X magnification under oil immersion after Gram Staining. Further, 24 h bacterial culture was centrifuged at 8000 rpm for 15 min, and the resulting cell pellet was washed twice, first with sterile distilled water and then with graded ethanol concentrations to ensure proper dehydration. The prepared cells were then carefully spread onto a sterile coverslip, air-dried at room temperature, and mounted for SEM imaging. The analysis was performed using a Zeiss EVO 18 Cryo SEM (Jena, Germany) operating at an accelerating voltage of 15 kV (Gangaraju et al., 2014; Haddad et al., 2022).

#### 2.4.2 Biochemical identification

Bacterial colonies exhibiting characteristic morphological features were subjected to further biochemical confirmation using the potassium hydroxide (KOH) solubility test and the catalase test. Catalase activity was assessed by transferring a single, well-isolated colony onto a clean glass slide and exposing it to atmospheric oxygen for a short duration. Subsequently, 1–2 drops of 3% hydrogen peroxide (H₂O₂) were added to the colony. The immediate release of oxygen bubbles signified a catalase-positive reaction, whereas the absence of effervescence indicated a catalase-negative strain. Biochemical tests such as MR-VP test, Indole test, Citrate Utilization test, and fermentation patterns of different sugars, as well as morphological traits such as colony colour, shape size, elevation were analyzed (Augustine, 2024). All the tests were performed in triplicate, and the results were interpreted according to standard microbiological procedures. The bacterial culture was characterized based on their ability to ferment a range of different carbohydrates using K024-1 KT kit (HiMedia, Mumbai, India). The carbohydrates tested consist of D-Glucose, Lactose monohydrate, D-Maltose, Sucrose, *β*-D-Glucose, D-Ribose, D-Arabinose, D-Galactose, D-Mannose, D-Fructose, and D-Xylose. The bacterial culture was then preserved in MRS agar slant and stored at 4 °C for future studies.

### 2.5 Screening for GABA-producing bacteria

A 48-h bacterial culture was centrifuged (8,000 rpm for 15 min at 4 °C) to obtain the cell-free supernatant. Thin-layer chromatography (TLC) was performed using silica gel 60 plates (20 × 20 cm) for all 188 Gram-positive bacteria. An aliquot of 3 μL of the supernatant was carefully spotted onto the plates. 10 mg/mL of standard GABA and MSG was used as reference. The chromatograms were developed using a solvent system consisting of acetic acid, 1-butanol, and distilled water in a ratio of 1:4:5 (v/v/v). After development, the plates were air-dried and sprayed with 0.5% ninhydrin solution to detect amino compounds (Gangaraju et al., 2014). The presence of spots with respect to GABA standard was used for the identification of GABA. The selected GABA-positive cultures were subjected to High Performance Liquid Chromatography (HPLC) for quantification of GABA. In order to perform the analysis, a 10 μL aliquot of the cell-free supernatant was derivatized with 20 μL OPA-MCE reagent and 100 μL sodium borate buffer (0.4 M, pH 10.4) at 30 ± 2 °C for 5 min (Gangaraju et al., 2014). The mixture was filtered through a 0.22 μm syringe filter (Millipore, MA, USA) and analyzed using a Shimadzu LC-20 AD HPLC system with an SPD-20A UV detector. A 20 μL sample was injected into a Thermo Fisher analytical column. The mobile phase comprised triethylamine (200 μL) and sodium acetate (1.64 g) in 1000 mL of 20% (v/v) acetonitrile. The flow rate was 0.5 mL/min, and detection was performed at 338 nm at 30 ± 2 °C (Hui et al., 2016). To confirm the presence of GABA in the sample, we performed Gas chromatography–Mass Spectrometry (GCMS) in addition to HPLC.

Cell-free supernatant from the selected probiotic culture, incubated for 48 h in MRS broth supplemented with 3% MSG, was centrifuged (8,000 rpm for 15 min at 4 °C). The supernatant was filtered using 0.22 μm syringe filters (Millipore, MA, USA) and derivatized with ethyl chloroformate. Samples (50–200 μL) were diluted in chloroform for GC–MS analysis. Analysis was conducted using an Agilent 8,890 GC system coupled to a 5977C MSD, equipped with an HP-5MS UI column (30 m × 250 μm × 0.25 μm). Injections (0.5 μL) were made in splitless mode at 260 °C. The oven temperature was initially held at 120 °C for 2 min, then increased at a rate of 20 °C/min to 300 °C, and maintained at this final temperature for 10 min, resulting in a total run time of 21 min. The MS was operated in electron ionization (EI) mode, with the quadrupole temperature set to 150 °C. Data acquisition was conducted in Selected Ion Monitoring (SIM) mode, using helium as the carrier gas at a constant flow rate of 1.2 mL/min (Michlig et al., 2024).

### 2.6 Differential GABA production in complex and minimal media

MRS media with five different compositions were considered for evaluation. The composition and pH of these media are given in Table 1 (Gangaraju et al., 2014). 24 h culture of S15-3, incubated at 37 °C was inoculated in 50 mL of various media in 250-mL Erlenmeyer flasks and again incubated for 48 h. The changes in pH were noted, and quantification of GABA production was measured.

**Table 1:** MRS media with five different compositions.

Medium	Components (g/L)	Initial pH	References
MRS1	MRS broth: 55.15; MSG: 20	6.8	Ratanaburee et al. (2011)
TYG	Yeast extract: 5; Dextrose: 10; Tryptone: 5; MSG: 20	7.0	Li et al. (2008)
GYP	Proteose peptone: 5; Yeast extract: 10; Dextrose: 10; Sodium acetate: 2; Magnesium sulfate: 0.2; Manganese (II) sulfate tetrahydrate: 0.01; Ferrous sulfate heptahydrate: 0.01; MSG: 20	7.0	Gangaraju et al. (2014)
MRS2	MRS broth: 55.15; MSG: 50	6.8	Gangaraju et al. (2014)
Modified MRS	Yeast extract: 6; Dextrose: 25; Polysorbate: 1; Tryptone: 6; Magnesium sulfate: 0.2; Manganese (II) sulfate tetrahydrate: 0.05; MSG: 20	6.8	Gangaraju et al. (2014)

### 2.7 Molecular identification of the best GABA-producing isolates

The top GABA-producing isolate was identified by 16S rRNA gene sequencing. Genomic DNA was extracted and assessed on a 1% agarose gel. The 16S rRNA gene was amplified using universal primers 27F and 1492R, and the PCR product was purified. Sanger sequencing was performed using primers 785F and 907R on an ABI 3730xl Genetic Analyzer with the BDT v3.1 Cycle Sequencing Kit. Forward and reverse reads were assembled into a consensus sequence using the aligner software. BLAST analysis was conducted against the NCBI ‘nr’ database to identify closest matches. The top ten hits were aligned using Clustal W, and a phylogenetic tree was constructed using the Maximum Likelihood method in MEGA11 (Sumalata, 2023). The final sequence was submitted to the NCBI database.

### 2.8 Biological characteristics and evaluation of probiotic attributes

A microorganism is considered as a probiotic when it fulfills specific functional criteria that enable it to confer health benefits to the host. The probiotic potential of the top GABA-producing isolate, *Enterococcus faecalis* RIT BM_S15-3_ (S15-3) was evaluated based on its tolerance to gastrointestinal stress conditions, including acid tolerance, bile salt tolerance, and survivability under simulated gastrointestinal conditions.

#### 2.8.1 Acid and bile tolerance

The isolate S15-3 (10% v/v) was cultured in MRS broth at 37 °C for 24 h. Cells were harvested by centrifugation (8,000 rpm, 15 min, 4 °C) and resuspended in fresh sterile MRS broth. For acid tolerance, the pH was adjusted to 1.5, 2.0, and 3.0 using 5 N HCl. Viability was assessed at 0, 90, and 180 min using the spread plate method, and results were expressed as log CFU/mL (Chen D. et al., 2023). For bile salt tolerance, MRS broth was supplemented with oxgall at 0.15, 0.3, and 0.45%, inoculated with S15-3 (10% v/v), and incubated at 37 °C for 24 h. Viable counts were determined at 0, 12, and 24 h on MRS agar and expressed as log CFU/mL (Chen Z. et al., 2023).

#### 2.8.2 Viability under simulated gastrointestinal conditions

The survivability of isolate S15-3 in simulated gastrointestinal conditions was assessed after sequential exposure to salivary, gastric, and intestinal fluids. S15-3 (10% v/v) was first incubated with salivary *α*-amylase (100 U in 0.1 M sodium acetate buffer, pH 5.6) at 37 °C for 2 min (Gangaraju et al., 2014). Viable cells were then exposed to simulated gastric fluid (SGF; pH 2.0) containing a sterile food suspension [starch (3 g/L), pectin (2 g/L), mucin (4 g/L), glucose (0.4 g/L), yeast extract (3 g/L), peptone (1 g/L), and cysteine-HCl (0.5 g/L)] and incubated at 37 °C for 2 h. Post-incubation, cells were harvested, washed with 0.2 M phosphate buffer (pH 7.0), and transferred into simulated intestinal fluid (SIF), composed of SGF supplemented with 0.5% oxgall and 2.5 g/L sodium bicarbonate (pH 6.5), followed by incubation at 37 °C for 16 h. The intestinal tolerance of S15-3 was further evaluated by exposing the cells to pancreatic enzymes such as trypsin, amylase and lipase (Chen D. et al., 2023). Briefly, the overnight culture of S15-3 was harvested by centrifugation at 8000 rpm for 15 min at 4 °C. The cell pellet was washed twice with sterile phosphate-buffered saline (PBS, pH 7.2) and resuspended to obtain an approximate cell density of 10^8^ CFU/mL. 10% (v/v) of the standardized bacterial suspension was mixed with SIF containing pancreatic enzymes and incubated at 37 °C for 3 h. A control sample without pancreatic enzymes was maintained under identical conditions. At predetermined time intervals (0 h and 3 h), samples were withdrawn, serially diluted in sterile saline, and plated on MRS agar. The plates were incubated at 37 °C for 24–48 h, and viable cell counts were then determined. Survival under simulated intestinal conditions was expressed as log CFU/mL and calculated by comparing viable counts before and after enzyme exposure.

Survival (%) = $\frac{\mathrm{CFU}\;\text{after enzyme treatment}}{\mathrm{CFU}\;\text{of control}}\times 100.$

#### 2.8.3 Antibiogram

The antibiotic susceptibility of 24 h S15-3 culture grown in MRS broth at 37 °C was assessed using the disc diffusion method (Gaur et al., 2023). A 100 μL aliquot of the culture was spread onto MRS agar plates using a sterile L-rod. Commercial antibiotic discs were placed on the agar surface, and plates were incubated at 37 °C for 24 h. Zones of inhibition were measured to determine susceptibility (Kim et al., 2022). The antibiotics and their concentrations (μg/disc) included: azithromycin (30), vancomycin (30), ciprofloxacin (15), amikacin (15), linezolid (15), tetracycline (30), rifamycin (5), erythromycin (15), penicillin-G (10), and ampicillin (10).

#### 2.8.4 Antimicrobial activity

The antimicrobial activity of *Enterococcus faecalis* RIT BM_S15-3_ was assessed against *Staphylococcus aureus* MTCC 96, *Bacillus cereus* MTCC 441, *Escherichia coli* MTCC 42, and *Pseudomonas aeruginosa* MTCC 424, following the method of Gangaraju et al. (2014) with minor modifications. *Enterococcus faecalis* RIT BM_S15-3_ was cultured in MRS broth at 37 °C for 24 h, and the cell-free supernatant was obtained by filtration through a 0.22 μm PTFE membrane filter (Millipore, India). The pH was adjusted to 6.8 using 0.1 M NaOH. Pathogenic strains were grown in nutrient broth at 37 °C for 18 h, and 10^5^ CFU/mL of each was spread on Mueller-Hinton (MH) agar plates. Wells (6 mm diameter) were punched using a sterile cork borer and filled with 25, 50, 75, 100, 125, and 150 μL of the neutralized supernatant. 100 μL of 0.05 mg/mL Ciprofloxacin was used as positive control and deionized water as negative control. Plates were incubated at 37 °C for 24 h, and antimicrobial activity was determined by measuring the diameter of the inhibition zones.

### 2.9 Hemolysis and gelatinase activity

The safety of *Enterococcus faecalis* RIT BM_S15-3_ was evaluated by assessing hemolytic and gelatinase activity. For hemolysis, S15-3 was cultured in MRS broth at 37 °C for 24 h and streaked onto blood agar plates containing 5% defibrinated sheep blood. Plates were incubated at 37 °C, and hemolytic activity was interpreted based on zone appearance: greenish discoloration (*α*-hemolysis, indicative of opportunistic pathogens), clear zones (*β*-hemolysis, associated with pathogenicity), and no zone (*γ*-hemolysis, indicating non-pathogenicity) (Lad et al., 2022). For gelatinase activity, S15-3 was cultured into MRS broth at 37 °C for 24 h and streaked onto MRS agar supplemented with 3% gelatin. Plates were incubated at 37 °C for 24 h and gelatinase activity was assessed by zone of clearance. The appearance of a clear zone surrounding the microbial growth served as an indicator of positive gelatinase activity and absence of zone, for negative gelatinase activity (Abdelrahman et al., 2024).

### 2.10 Optimization of culture conditions for GABA production

#### 2.10.1 Effect of substrate concentration on GABA production

The influence of substrate concentration on GABA production was evaluated as described by (Sharma et al., 2022) with slight modifications. Briefly, 50 mL of MRS broth supplemented with varying concentrations of MSG (1, 2, 3, 4, 5, 7, and 10%) was taken in different conical flasks and inoculatedwith a 24 h culture of S15-3 and incubated under static fermentation conditions at 37 °C. Initial pH was adjusted to 6.8. 1 mL samples were collected from each flask at regular interval and the biomass and GABA content obtained were quantified using HPLC.

#### 2.10.2 Effect of temperature on GABA production

The influence of incubation temperature on GABA production over fermentation time was evaluated at various temperatures (30, 35, 37, 40, 45, and 50 °C), as described by Sharma et al. (2022). Briefly, 50 mL of MRS broth supplemented with 3% MSG was inoculated with a 24 h S15-3 culture, and the initial pH was adjusted to 6.8. The cultures were incubated under static conditions with specified temperatures, and 1 mL samples were collected at regular intervals. Biomass and GABA concentrations were quantified using HPLC.

#### 2.10.3 Effect of pH on GABA production

The effect of initial medium pH on GABA production was investigated over the course of fermentation, following the method of Sharma et al. (2022). MRS broth (50 mL) supplemented with 3% MSG was inoculated with a 24 h-culture of isolate S15-3. The initial pH was adjusted to values ranging from 2.0 to 8.0 Cultures were incubated under static conditions at 37 °C, and 1 mL aliquots were sampled at regular time intervals. Biomass accumulation and GABA production were quantified using HPLC. Growth (OD @ 600 nm) and GABA production were monitored over time and under varying environmental conditions, including pH, temperature, and substrate concentration. Detailed profiles are provided in the Supplementary material.

## 3 Results and discussion

### 3.1 Isolation of potential probiotic isolates

A total of 188 potential probiotic isolates were isolated from 17 human breastmilk samples.

### 3.2 Identification of potential probiotics

**Figure 1:**
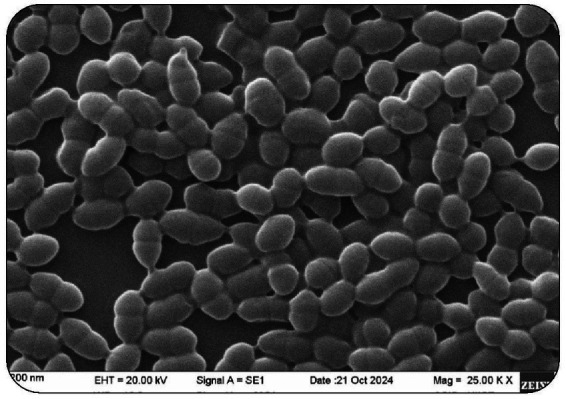
SEM image of probiotic diplococci isolated from human breast milk.

Inorder to identify the lactic acid bacteria, biochemical assays on the isolates were performed (Taye et al., 2021). The preliminary identification of the isolated cultures was conducted through morphological and biochemical analyses, including Gram staining, EM, MR-VP, H_2_S, indole production, citrate utilization, urease production, and the catalase test (Table 2). These tests were performed preliminarily to identify the isolates based on their biochemical characteristics. Gram staining revealed that over 90% of the isolates were Gram-positive cocci, while the remaining ~10% exhibited a rod-shaped (bacillary) morphology. These findings provided initial insights into the cellular structure and classification of the isolates. To gain detailed surface and structural characteristics, selected isolates were subjected to SEM. SEM analysis of the predominant isolate revealed diplococcal morphology at a magnification of 25.00 KX and a working distance of 12.5 mm (Figure 1). The observed morphology closely resembled that of *E. faecalis* as reported in the high-resolution FE-SEM micrographs of a previous study by Kim et al. (2020). However, SEM analysis was used only for morphological assessment and does not provide species-level identification. Definitive taxonomic identification of the isolate was achieved through 16S rRNA gene sequencing. Further, biochemical confirmation was performed using catalase activity assay, which help to differentiate Gram-positive from Gram-negative bacteria. In the catalase test, the isolates did not produce effervescence upon the addition of H₂O₂, indicating the absence of catalase enzyme activity and classifying them as catalase-negative. This result suggests the inability of the isolates to detoxify H₂O₂ into oxygen and water, a characteristic trait of many Gram-positive lactic acid bacteria. These biochemical findings were in agreement with previous studies, including that of Al Atya et al. (2015), which reported similar characteristics in probiotic bacterial isolates. In IMViC, H_2_S and urease tests performed, only Methyl Red test was positive for the isolate. It was tested negative for Indole, Voges–Proskauer (VP), Citrate utilization, urease activity, hydrogen sulfide (H₂S) production, and catalase activity.

**Table 2:** Biochemical test.

Test	Observation	Result
Indole	No red ring formation	Negative
Methyl Red	Red colour	Positive
Voges-Proskauer	Yellow	Negative
Citrate utilization	Yellow	Negative
Urease	Yellow	Negative
Catalase	No effervescence	Negative
H_2_S	No change of colour	Negative

The positive Methyl Red reaction indicates that stable acidic end products are produced during the fermentation of glucose, indicating a mixed-acid fermentative pathway (Khushboo et al., 2023). This observation is further supported by the negative VP result, which verifies that neutral end products such as acetoin are absent. The overall MR-VP profile demonstrates the dominance of acidogenic metabolism. The inability to use citrate as a sole carbon source and limited tryptophan breakdown are characteristics frequently linked to fermentative Gram-positive bacteria, respectively, as indicated by negative indole and citrate utilization results. The lack of urease activity suggests that the isolate has restricted nitrogen metabolism through urease-dependent pathways since it does not hydrolyze urea to ammonia. Further, carbohydrate fermentation was performed to evaluate the metabolic versatility and fermentative capabilities of the isolate. The study investigated the sugar utilization patterns of 11 different sugars of the isolate. The results (Table 3) indicated that the isolate was positive for D-Glucose, Lactose monohydrate, D-Maltose, Sucrose, *β*-D-Glucose, D-Arabinose, D-Galactose, D-Mannose, and negative for D-Xylose, D-Fructose and D-Ribose. Overall, these findings provide valuable insights into the sugar utilization capabilities of LAB and their potential as probiotic agents (Hamed, 2021).

**Table 3:** Carbohydrate utilization test.

Sl No	Carbohydrate	Reaction
01	D-Glucose	+
02	Lactose monohydrate	+
03	D-Maltose	+
04	Sucrose	+
05	Β-D-Glucose	+
06	D-Ribose	−
07	D-Arabinose	+
08	D-Galactose	+
09	D-Mannose	+
10	D-Fructose	+
11	D-Xylose	−

### 3.3 Screening for GABA-producing probiotics

**Figure 2:**
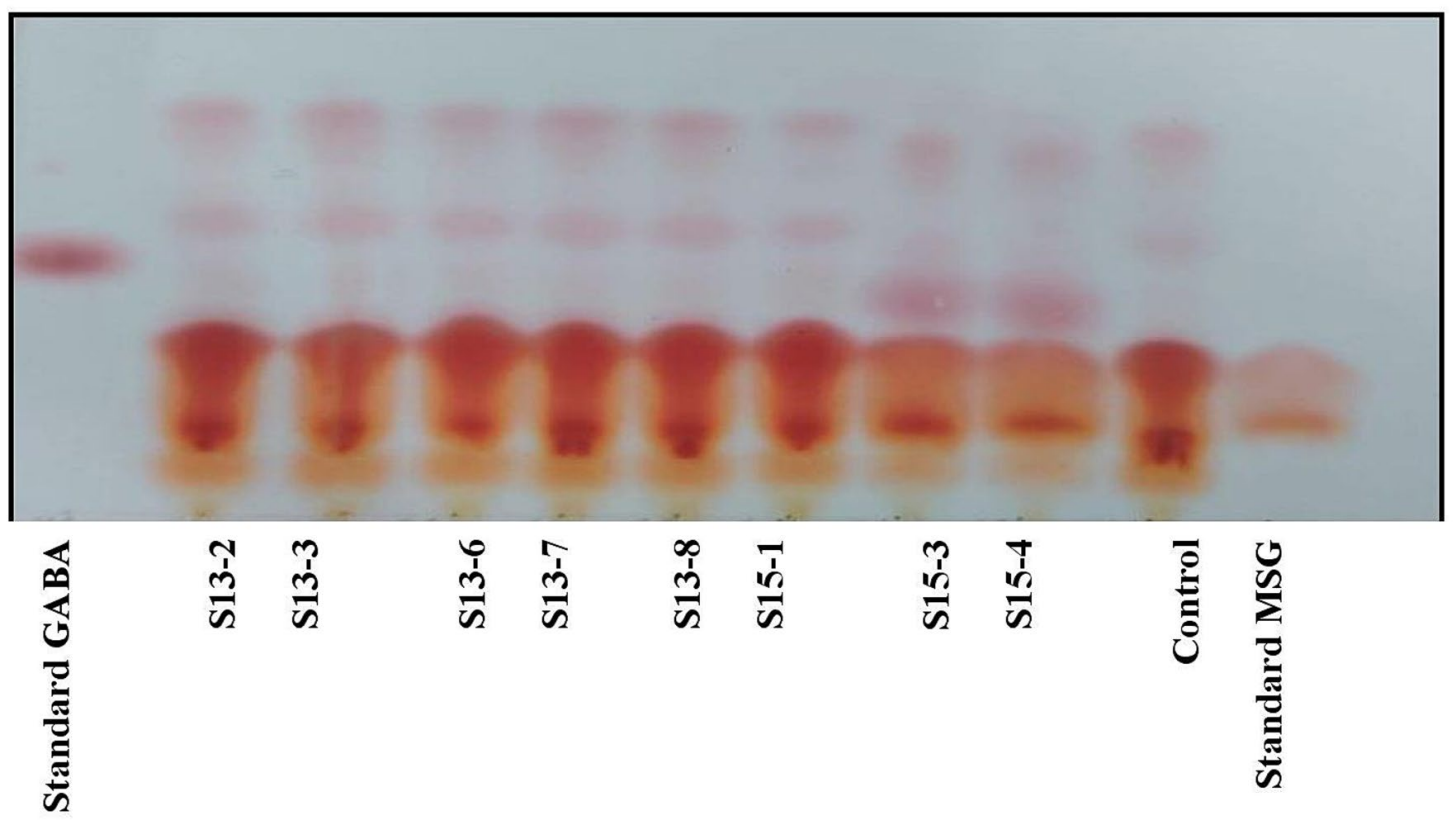
Thin layer chromatography of different breast milk isolates (Control-MRS media, 10 mg/mL of standard GABA and standard MSG).

GABA is the key inhibitory neurotransmitter present in the central nervous system of the human body, widespread within the hippocampus, brain’s cerebral cortex, as well as the cerebellum (Benarroch, 2021). In extracellular fluids, GABA normally exists between 0.5-2 mM for the purpose of balancing neuron excitability with that of the excitatory neurotransmitter glutamate (Pressey et al., 2023). GABA’s role in the preservation of mental stability has experienced an explosion of new interest that also aims at improving its availability by treatment as well as by the consumption of probiotics. 188 isolates were obtained from 17 breastmilk samples of which more than 90% were Gram-positive cocci, consistent with earlier findings of lactic acid bacteria being a predominant part of breastmilk microbiota (Gill, 2023). Screening and selection of potent GABA-producing probiotics were based on the qualitative analysis of GABA, by TLC. 3 μL of each sample was spotted on the TLC plate, and MRS broth was taken as a control. 10 mg/mL of standard GABA and MSG was used for the experiment. Among 188 isolates, 41 isolates tested positive as GABA was detected qualitatively. Among them, two of the isolates (S15-3 and S15-4) showed intense spots for GABA production, as shown in Figure 2. The intensity of the visualized spots varied, indicating that GABA production depends on isolate variation. All GABA suspected isolates were then subjected to HPLC analysis for quantification. GABA production was qualitatively analyzed using TLC, and similar results were obtained from a previous study where GABA was detected in a probiotic strain of *E. faecium* isolated from a fermented food (Gangaraju et al., 2014).

**Figure 3:**
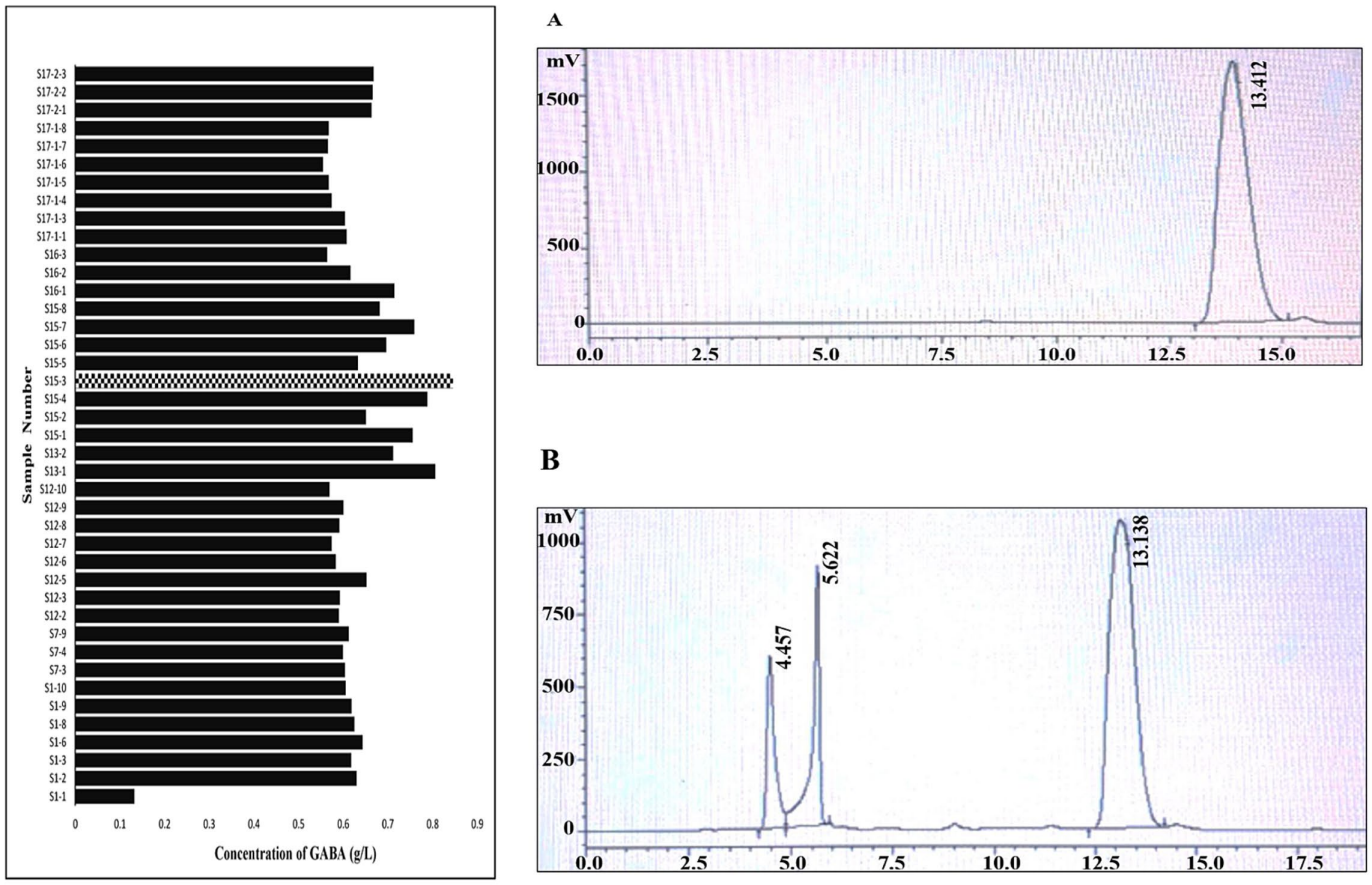
HPLC analysis of GABA positive samples. Chromatograms of standard GABA **(A)** at retention time of 13.712 and chromatogram of S15-3 **(B)** at retention time of 13.038 min. The *X* axis represents time in mins and the *Y* axis represents the electrical signal voltage output generated by the detector (mV).

The result of the TLC analysis was further confirmed by analyzing the cell supernatant A total of 188 isolates recovered from 17 breast milk donors were screened for GABA production, of which 41 isolates (21.8%) demonstrated detectable GABA synthesis (Figure 3). As isolate recovery varied among donors, prevalence was calculated relative to the total isolates obtained from each individual, ensuring appropriate donor-level normalization. The proportion of GABA-producing isolates per donor ranged from 0 to 40%, with several donors exhibiting moderate prevalence while others showed minimal or no detectable producers, indicating clear inter-individual variability in psychobiotic potential. Isolates from 8 donors failed to produce GABA even after supplementing the substrate. The high-producing strains were clustered within specific donor S15 (S15-3 and S15-4) and S13 (S13-1, S13-2). On an average, approximately 10 morphologically distinct colonies were selected per donor to ensure representative sampling. However, in 3–4 donors, more than 10 isolates were included due to the presence of well-defined colonies exhibiting distinct morphological characteristics (e.g., size, elevation, and margin), thereby increasing the total isolate count. This approach ensured adequate representation of phenotypic diversity while maintaining balanced sampling across donors.147 isolates turned out to be GABA negative. The concentration of GABA quantified for the positive strains is given in the graph below. The TLC results showed similar intensity for the isolates S15-3 and S15-4. In HPLC analysis, S15-3 produced 0.847111 g/L (8.45 mM), which is the highest concentration of GABA produced among positive isolates, and S15-4 produced 0.78971 g/L (7.66 mM). (Figure 3). The least GABA concentration of 0.13343 g/L (1.29 mM) was from sample number S1-1. The chromatogram from HPLC of cell supernatants of GABA-producing isolates (Figure 3B) showed a peak with a retention time (13.138 min) similar to that of GABA standard (Figure 3A) (13.412 min). Recent findings suggest that a probiotic strain capable of producing ≥1 mM of GABA in culture supernatant is considered promising for functional food and psychobiotic applications (Ibrahim et al., 2024). This result suggests that the isolate S15-3 producing 8.45 mM is significantly high and well within the functional range. The concentration of GABA produced from S15-3 isolated from human breastmilk aligns with the findings by Gangaraju et al. (2014), in which the probiotic bacteria were isolated from fermented food products. Further, the results from HPLC were confirmed by subjecting them to GCMS analysis as well.

**Figure 4:**
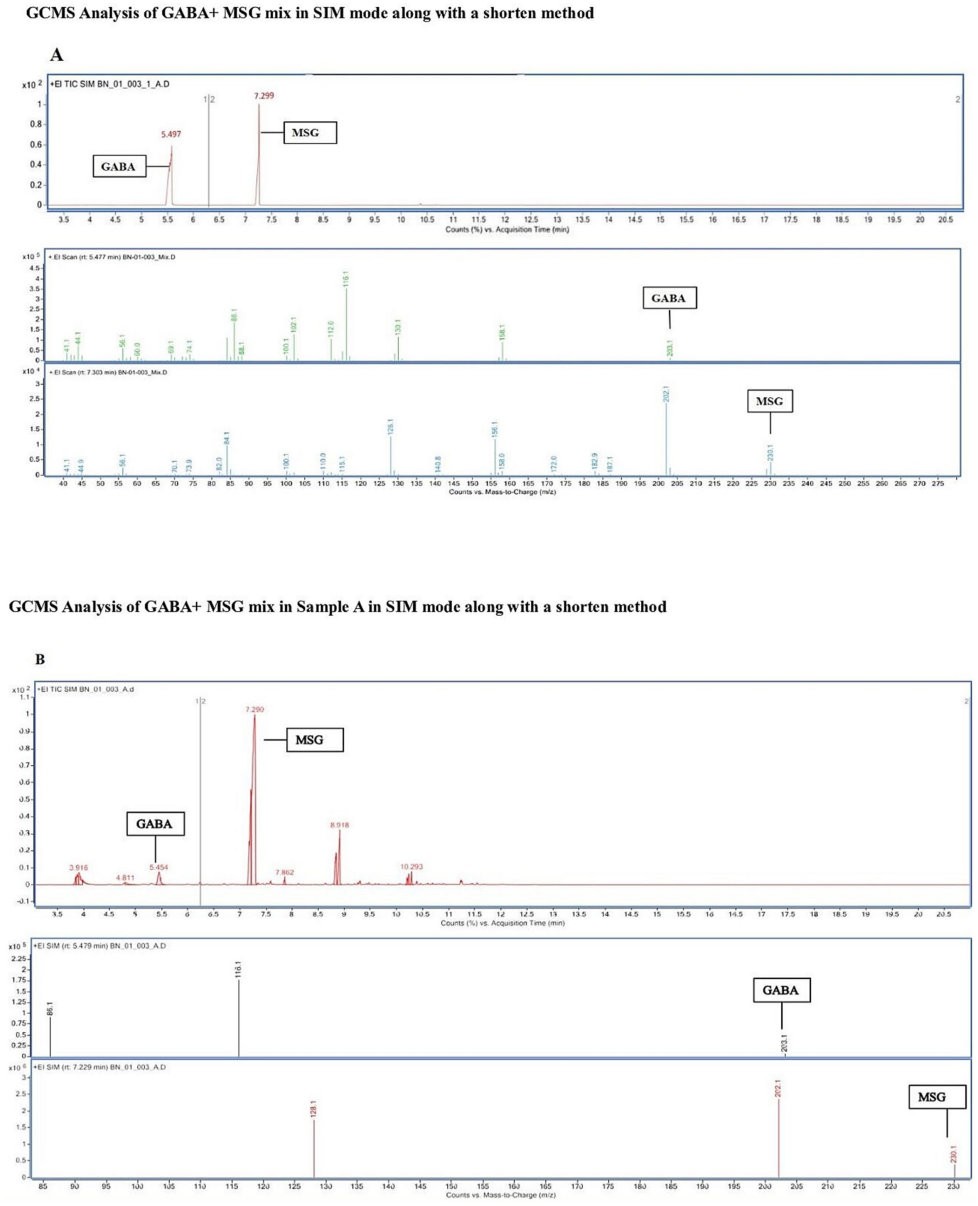
Chromatogram of counts vs. acquisition time (minutes) of GABA and MSG mix and mass spectra of counts (%) vs mass-to-charge showing **(A)** Standard GABA and MSG respectively. **(B)** Cell supernatant of probiotic strain after 48 h incubation.

The cell-free supernatant of S15-3 was filtered through a 0.22 μm membrane. Before analysis, chemical derivatization was performed to enhance compound detection, specifically converting carboxylate groups into ethyl esters and amines into ethyl carbonates (Zhang et al., 2025). This process modified GABA to a derivative with a molecular weight of 203 Da, eluting at approximately 5.497 min, while the substrate monosodium glutamate (MSG) was converted to a derivative of 230 Da, eluting at around 7.299 min in SIM mode. The ionization used in this analysis was electron ionization (EI). In EI, ion formation occurs through the removal of an electron, rather than the addition of a proton. Therefore, the molecular ion corresponds to the neutral molecular weight of the derivatized compound. Since derivatized GABA has a molecular weight of 203, the observed molecular ion at m/z 203 is consistent with electron ionization (EI) and does not involve the formation of a [M + H]^+^ species (Eskandari et al., 2023). Figure 4A represents the detection of standard GABA and MSG. The presence of GABA in the supernatant was confirmed by Figure 4B. The chromatogram displays counts versus m/z (mass-to-charge) ratio.

### 3.4 Differential GABA production of S15-3 in minimal and complex media

GABA production in S15-3 was found to be highest in MRS medium supplemented with 5% monosodium glutamate (MSG), referred to as MRS2. The nutrient stress conditions in MRS2 appear to stimulate the cells to efficiently utilize MSG and other available nutrients for GABA biosynthesis (Alkay et al., 2025). Slightly lower GABA yields were recorded in MRS1 and TYG media, whereas no GABA production was detected in GYP and modified media formulations. This suggests that while growth-promoting components are necessary, they alone do not fully support GABA synthesis; rather, the concentration of MSG as a substrate plays a critical role. The conversion of MSG to GABA occurs via the glutamate decarboxylase (GAD) enzyme present in the probiotic strains. Among the different concentrations tested, MRS medium with 3% MSG yielded the highest GABA production of 0.320 g/L (3.105 mM), followed closely by MRS with 2% MSG at 0.319 g/L (3.097 mM), and the modified medium at 0.300 g/L (2.912 mM).

### 3.5 Molecular identification of the best GABA-producing isolate

**Figure 5:**
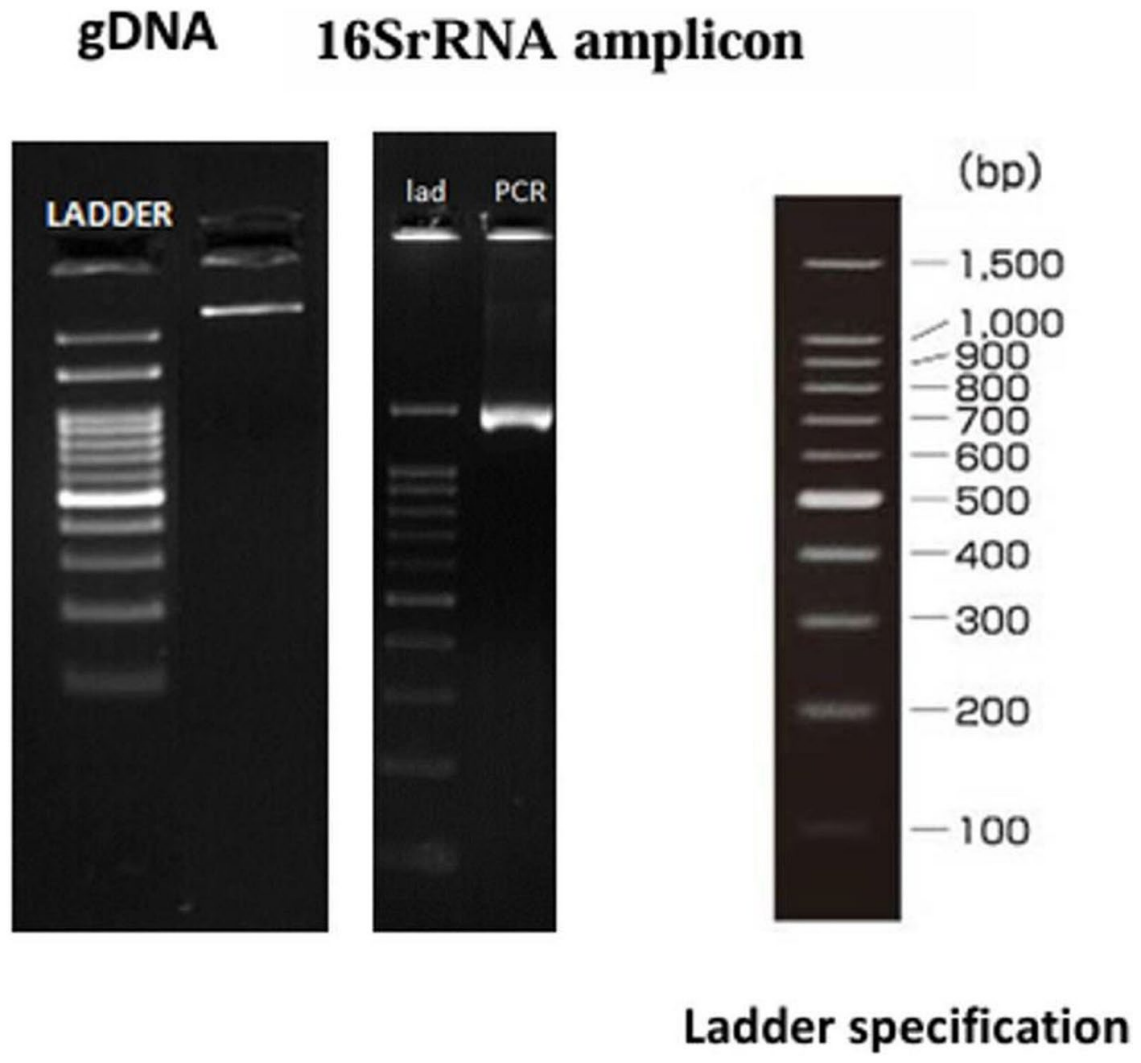
Amplification of 16S rRNA gene from genomic DNA.

**Figure 6:**
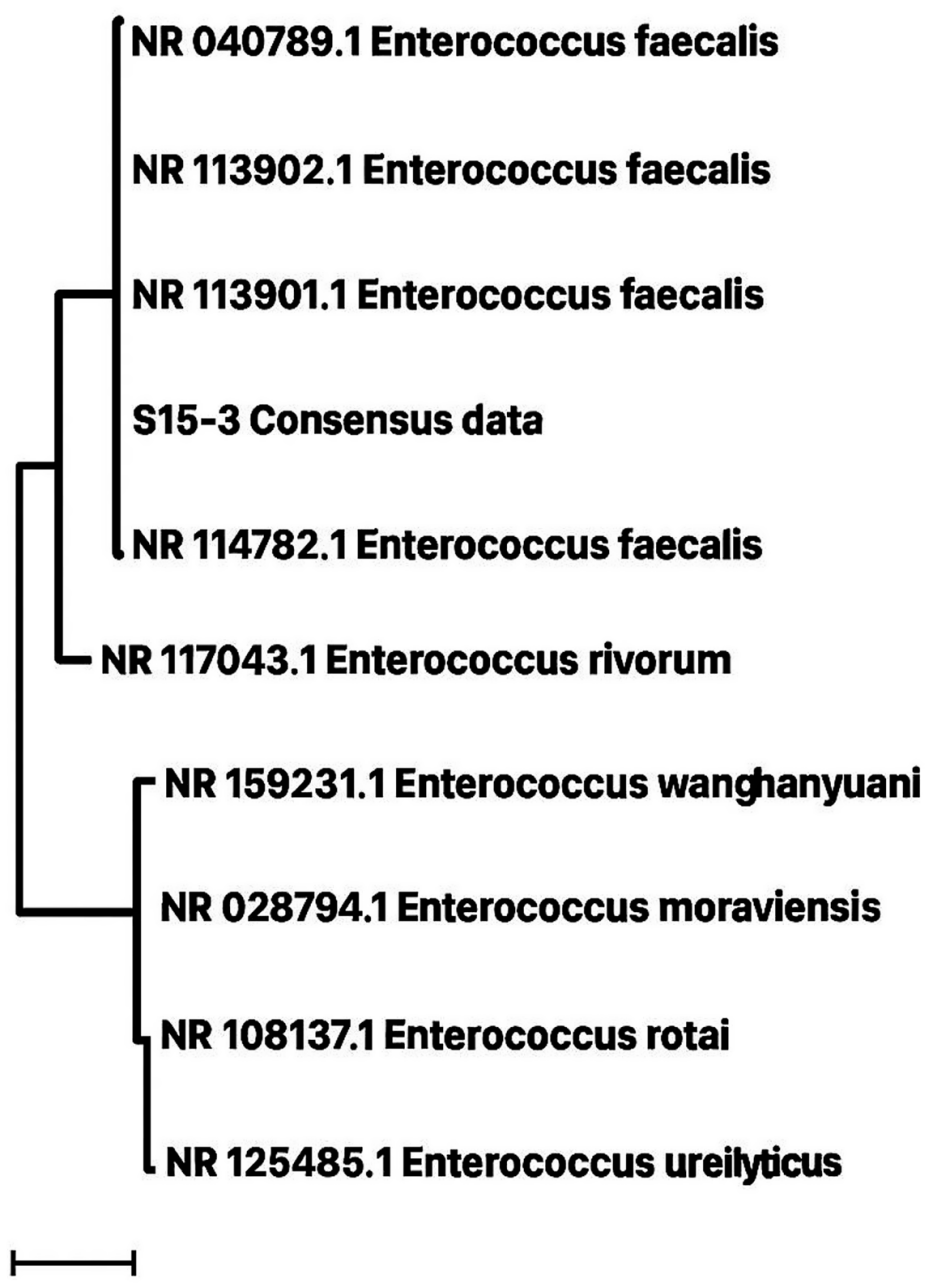
Taxonomical position of S15-3, *Enterococcus faecalis* RIT BM _S15-3_ as depicted by an unrooted phylogenetic tree.

The top GABA-producing isolate (S15-3) was further characterized genotypically through 16 s rRNA sequencing. The obtained sequence was analyzed using the BLAST tool and compared with sequences available in the NCBI database. The isolate showed the highest sequence identity of 99.93% (Table 4) with *E. faecalis* (Kim et al., 2020). Based on nucleotide homology and phylogenetic analysis (Figure 5), isolate S15-3 was identified as *E. faecalis*. Evolutionary analysis performed using the maximum likelihood method further supported this identification (Figure 6). Accordingly, the strain was designated as *Enterococcus faecalis* RIT BM_S15-3_ and the 16S rRNA gene sequence of isolate was deposited in the GenBank database under accession number PZ055568. Our approach focused on isolating phenotypically distinct colonies and evaluating their GABA-producing capacity, followed by 16S rRNA identification of the top-performing strain. Whole-genome sequencing, which is not done in this study, would offer more precise species-level resolution and insights into probiotic and safety-related genes, even though 16S rRNA sequencing is a reliable preliminary method.

**Table 4:** Blast sequence results showing similarity of organisms with S15-3.

Description	Scientific name	Max score	Total score	Query cover	E value	Per identity	Acc Len	Accession
*Enterococcus faecalis* strain LMG 7937 16S ribosomal RNA, partial sequence	*Enterococcus faecalis*	2,776	2,776	99%	0	99.74%	1,556	NR_114782.1
*Enterococcus faecalis* strain JCM 5803 16S ribosomal RNA, partial sequence	*Enterococcus faecalis*	2,752	2,752	99%	0	99.54%	1,517	NR_040789.1
*Enterococcus faecalis* strain ATCC 19433 16S ribosomal RNA, partial sequence	*Enterococcus faecalis*	2,734	2,734	97%	0	99.93%	1,483	NR_115765.1
*Enterococcus faecalis* strain NBRC 100481 16S ribosomal RNA, partial sequence	*Enterococcus faecalis*	2,628	2,628	93%	0	99.93%	1,426	NR_113902.1
*Enterococcus faecalis* strain NBRC 100480 16S ribosomal RNA, partial sequence	*Enterococcus faecalis*	2,628	2,628	93%	0	99.93%	1,426	NR_113901.1
*Enterococcus faecalis* strain S299 16S ribosomal RNA, partial sequence	*Enterococcus rivorum*	2,601	2,601	93%	0	98.38%	1,479	NR_117043.1
*Enterococcus wangshanyuanii* strain MN05 16S ribosomal RNA, partial sequence	*Enterococcus wangshanyuanii*	2,590	2,590	99%	0	97.56%	1,518	NR_159231
*Enterococcus rotai* strain CCM 4630 16S ribosomal RNA, partial sequence	*Enterococcus rotai*	2,566	2,566	98%	0	97.48%	1,514	NR_108137.1
*Enterococcus ureilyticus* strain CCM 4629 16S ribosomal RNA, partial sequence	*Enterococcus ureilyticus*	2,560	2,560	98%	0	97.41%	1,528	NR_125485.1
*Enterococcus moraviensis* strain 330 16S ribosomal RNA, partial sequence	*Enterococcus moraviensis*	2,547	2,547	99%	0	97.09%	1,509	NR_028794.1

### 3.6 Biological characteristics and evaluation of probiotic attributes

#### 3.6.1 Gastrointestinal stress tolerance

##### 3.6.1.1 Acid tolerance

**Figure 7:**
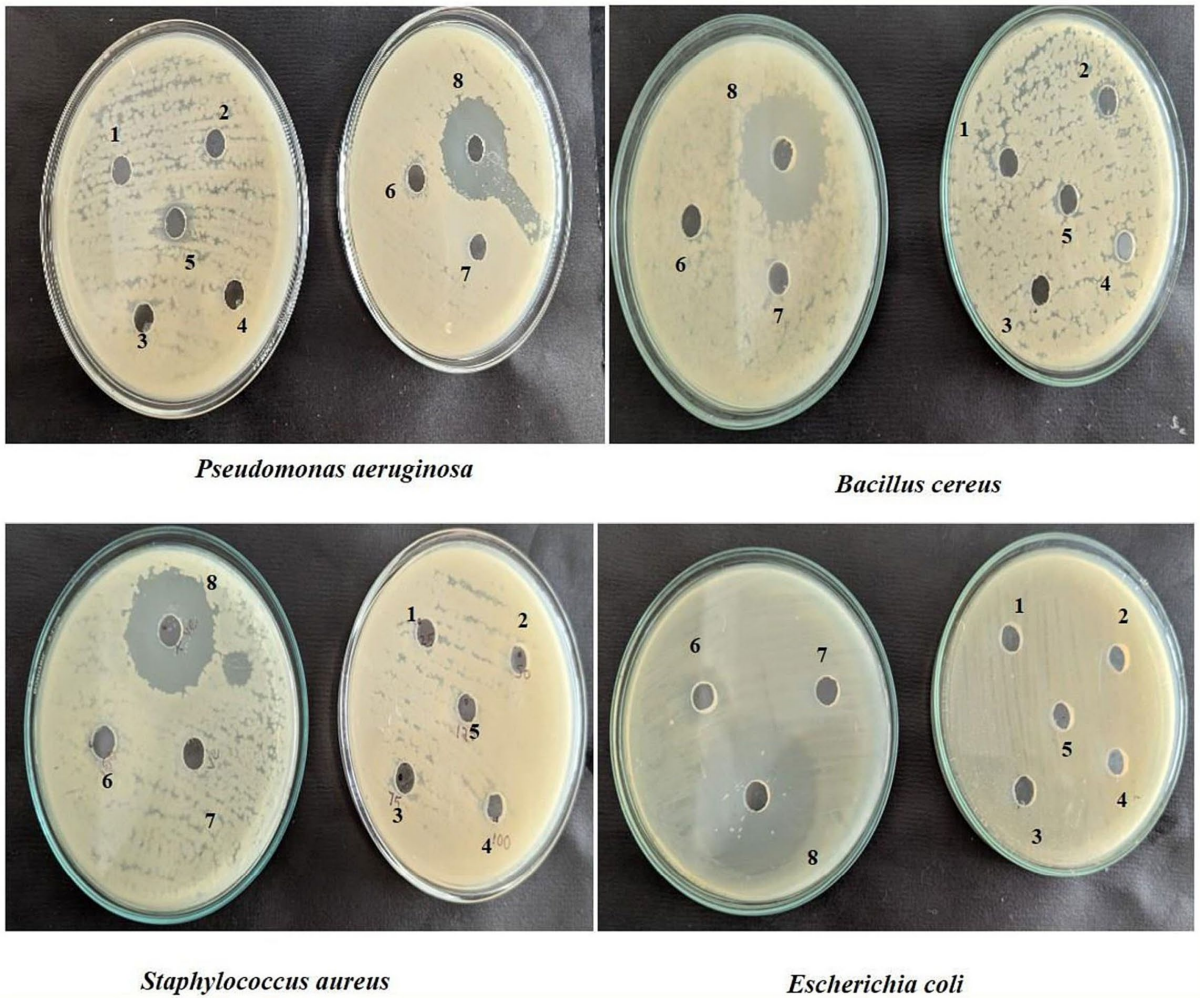
Antimicrobial activity of different concentrations of *E. faecalis* RIT BM _S15-3_ against Gram positive and Gram-negative pathogenic organisms. (1)25 μL (2) 50 μL (3) 75 μL (4) 100 μL (5) 125 μL (6) 150 μL (7) deionized water as negative control (8) Ciprofloxacin (100 μL).

**Figure 8:**
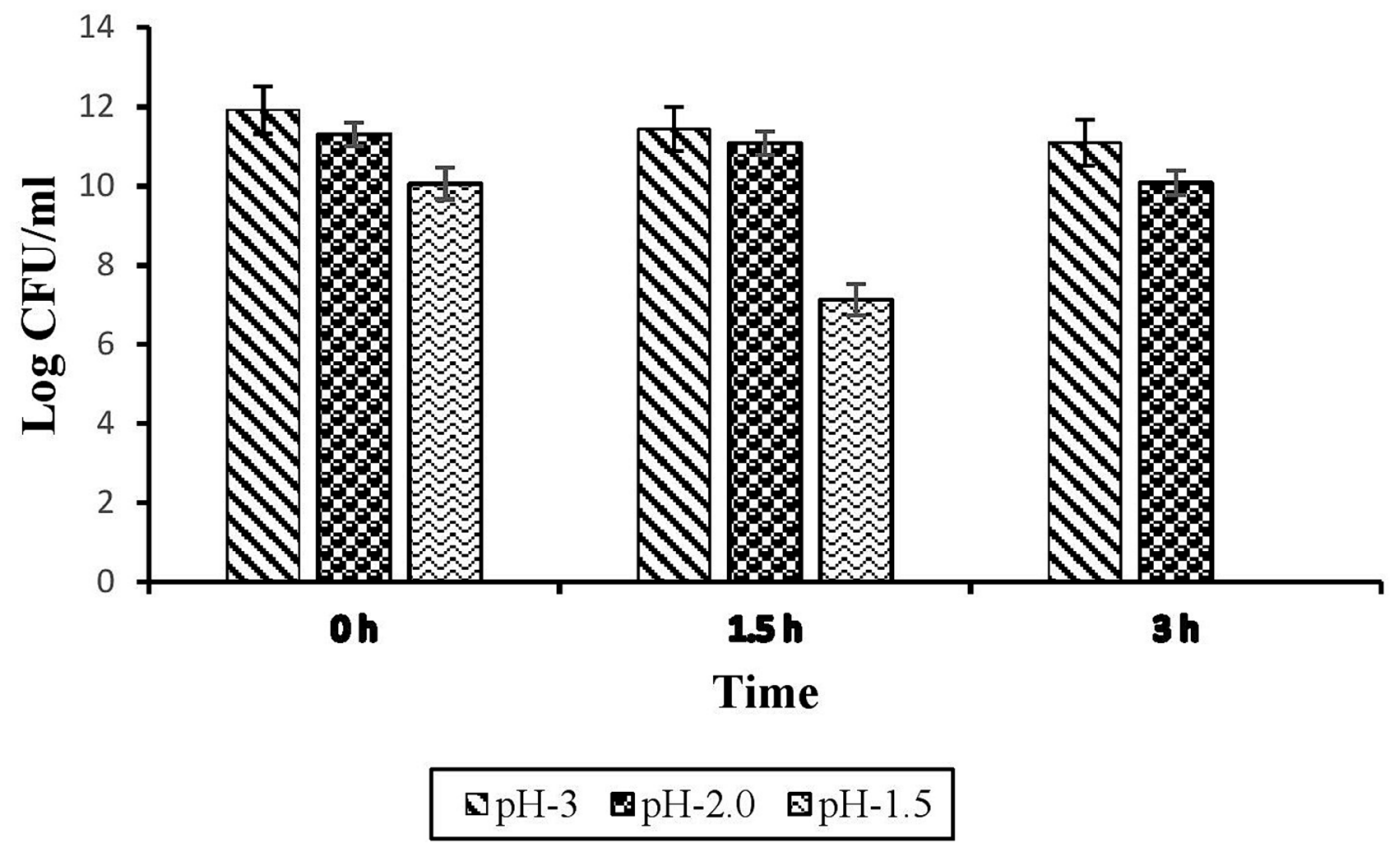
Acid tolerance of *Enterococcus faecalis* RIT BM _S15-3_ at different pH levels. Given data represents the mean of three separate experiments, and error bars represent standard error (SD).

*Enterococcus faecalis* RIT BM_S15-3_ demonstrated the ability to withstand acid stress across a range of pH values (1.5, 2.0, and 3.0) for up to 3 h. The results of the pH tolerance assay are illustrated in Figure 7. The highest viability was recorded at pH 3.0 (Figure 8), reaching 11.435 log CFU/mL after 1.5 h of exposure. The culture exhibited tolerance at pH 2.0 and 3.0, after 3 h of exposure, retaining viable counts of 11.09 and 10.08 log CFU/mL, respectively. At pH 1.5, the strain remained viable for up to 1.5 h; however, no viable cells were detected after 3 h of exposure at pH 1.5. These findings indicate that the isolate can withstand highly acidic conditions for a duration comparable to the typical gastric transit time suggesting its potential to survive passage through the stomach. Despite a slight decline in cell concentration, the strain exhibited notable acid tolerance at pH 2.0 and 3.0 over the 3 h. These findings are consistent with earlier reports; for instance, Shi et al. (2020) observed the tolerance of *E. faecium* MK-SQ-1 to acid, bile salts, trypsin and high temperature. The survival rate of the strain was nearly 90% after staying in the solution with pH 4.0 for 3 h and decreased to about 80% by staying in the solution with pH 3.0 for 3 h. These results demonstrate the strain’s ability to withstand gastric acidity, a crucial prerequisite for the functionality of probiotics. Evaluation of the probiotic characteristics involved robust acid tolerance (pH 2.0–3.0) which is a crucial index of survival throughout the gastrointestinal system. Such resistance corresponds favorably with previous reports of LAB species (Suharsono et al., 2023; Rashid et al., 2025).

##### 3.6.1.2 Bile tolerance

**Figure 9:**
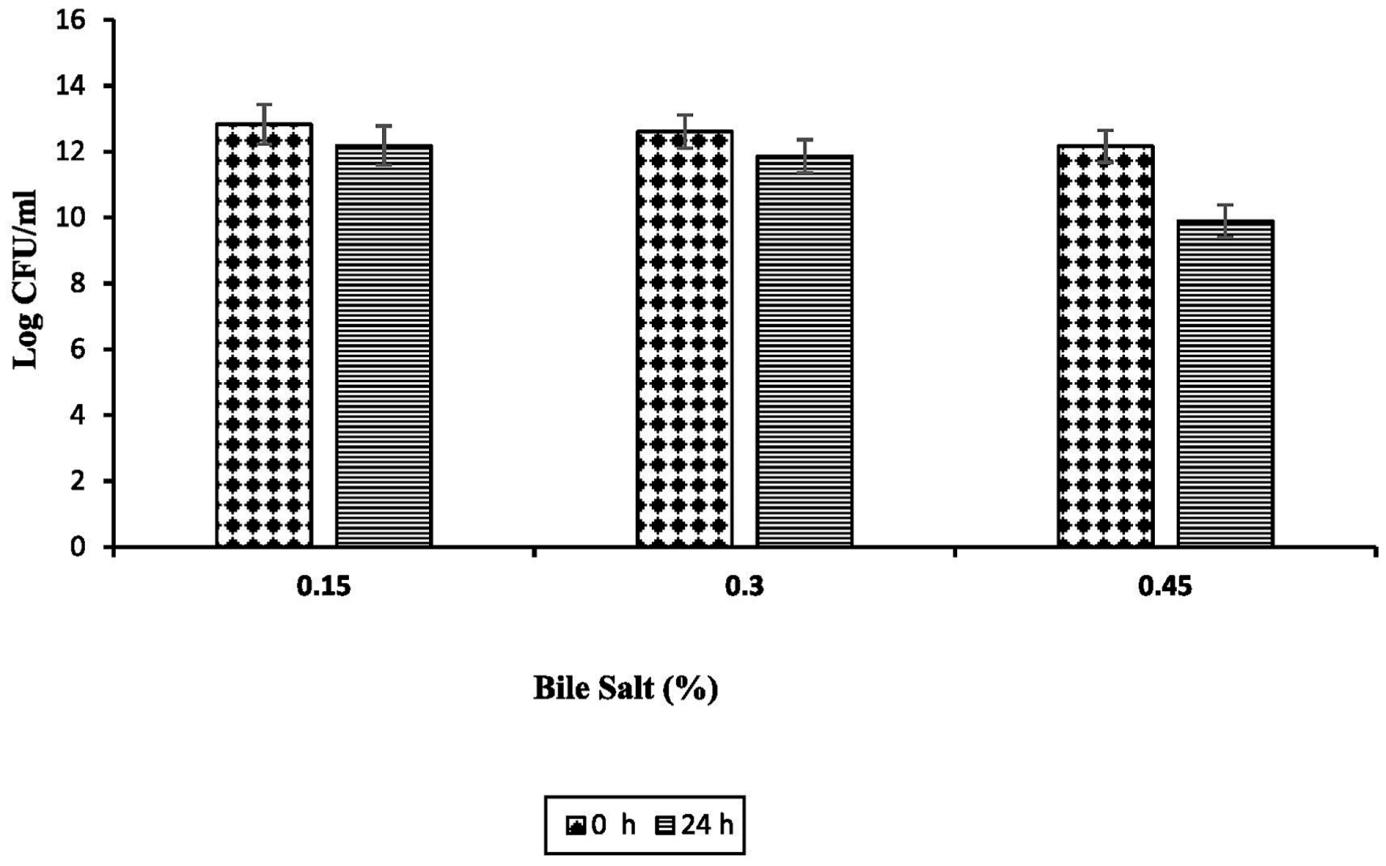
Bile salt resistance of *Enterococcus faecalis* RIT BM _S15-3_ at different bile salt solution. Given data represent the mean of three separate experiments, and error bars represent standard error (SD).

The bile salt tolerance of *Enterococcus faecalis* RIT BM_S15-3_ was evaluated by exposing the isolate to increasing bile salt concentrations (0.15, 0.3, and 0.45%) for 24 h. The viable cell counts after exposure were 12.18, 11.86, and 9.895 log CFU/mL at 0.15, 0.3, and 0.45% bile salts, respectively (Figure 9), indicating sustained survival even at elevated bile concentrations.

Resistance to bile salts is a primary prerequisite for the survival and colonization of probiotic bacteria in the gastrointestinal tract and is therefore considered a key determinant of probiotic potential (Toupal and Coşansu, 2024). A bile salt concentration of 0.3% is generally regarded as a critical threshold for evaluating bile tolerance in probiotic candidates (Rashid et al., 2025). Comparable tolerance has been reported for enterococci; for instance, *E. durans* was shown to survive at pH 3.0 and in the presence of bile salt concentrations up to 15 mg/mL (Comerlato et al., 2022). The ability of *E. faecalis* RIT BM_S15-3_ to maintain high viability across increasing bile concentrations may be attributed to adaptive mechanisms such as bile salt hydrolase activity, efflux pump systems, and maintenance of cell membrane integrity, collectively supporting its probiotic potential.

#### 3.6.2 Viability under simulated gastrointestinal conditions

**Figure 10:**
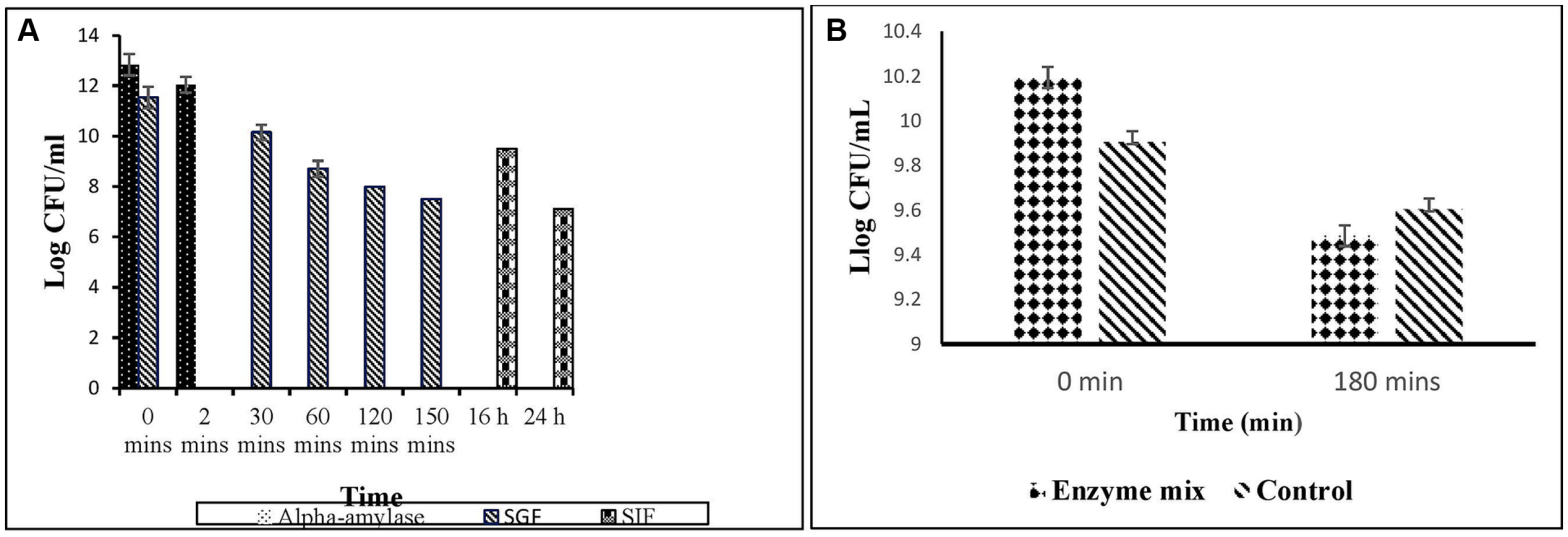
**(A)** Viability of *Enterococcus faecalis* RIT BM _S15-3_ under SGF conditions. Given data represents the mean of three individual experiments, and error bars represent standard error (SD). **(B)** Viability of *Enterococcus faecalis* RIT BM_S15-3_ under simulated intestinal enzyme conditions. Control is devoid of enzymes. Given data represents the mean of three individual experiments, and error bars represent standard error (SD).

The viability of *E. faecalis* RIT BM_S15-3_ under SGI is represented in Figure 10A. A significant effect was shown by alpha-amylase on the survivability of the organism. As time progressed, the viability of the culture decreased after incubation. It still survived stably until the end of treatment (24 h) with a viability of 7 × 10^3^ log CFU/mL. In simulated intestinal enzyme mix, the isolate showed a survivability of 9.485 × 10^8^ log CFU/mL after 3 h (Figure 10B). That is, 73.1% of the cells survived after enzyme treatment for 3 h, showing good viability in the intestine. This level of survivability is consistent with previously reported probiotic strains subjected to simulated gastrointestinal conditions. For instance, several lactic acid bacteria, including *Enterococcus* spp., have been shown to retain viability within the range of 6–8 log CFU/mL following simulated gastrointestinal transit (Vaccalluzzo et al., 2025). It is widely acknowledged that a minimum viable population of 10^6^–10^7^ CFU/mL is required to confer probiotic benefits in the host (Debnath and Miyoshi, 2024).

### 3.7 Antibiogram

The antibiotic susceptibility profile of the 24 h *E. faecalis* RIT BM_S15-3_ culture is presented in Table 5. The strain exhibited sensitivity to vancomycin, amikacin, linezolid, tetracycline, rifamycin, and ampicillin at the tested concentrations. This sensitivity suggests that the isolate is not a hospital-acquired, multidrug-resistant strain, thereby supporting its safety for probiotic and food-related applications. The observed susceptibility to these antibiotics suggests a favorable phenotypic safety profile; however, phenotypic sensitivity alone does not preclude the presence of silent or non-transferable resistance genes. Therefore, comprehensive genomic analyses would be required to conclusively rule out transferable resistance determinants and to fully assess the risk of horizontal gene transfer (Anifowose et al., 2024). However, the strain showed resistance to azithromycin, ciprofloxacin, erythromycin, and penicillin-G.

**Table 5:** Antibiogram of *E. faecalis.*

Antibiotic	Concentration (ug/mL)	Sensitivity (S)/Resistivity (R)
Azithromycin	30	R
Vancomycin	30	S
Ciprofloxacin	15	R
Amikacin	15	S
Linezolid	15	S
Tetracycline	30	S
Rifamycin	5	S
Erythromycin	15	R
Penicillin-G	10	R
Ampicillin	10	S

### 3.8 Antimicrobial activity

*Enterococcus faecalis* RIT BM_S15-3_ was found to be more effective against *Escherichia coli* MTCC 42 (7 mm diameter) and less effective against *Pseudomonas aeruginosa* MTCC 424 (3 mm diameter) at 125 μL and 150 μL, respectively (Figure 7). These differences are attributed to the structural differences between the organisms. Although both *E. coli* and *P. aeruginosa* are Gram negative, *P. aeruginosa* has a more robust outer membrane, which consists of high lipid content. In addition, low membrane permeability and efflux pumps make it intrinsically more resistant to antimicrobials (Islam et al., 2023). At this stage, the inhibitory activity observed cannot be specifically attributed to bacteriocins. Therefore, the antimicrobial effect is more appropriately described as being mediated by bacteriocin-like metabolites or other inhibitory compounds produced by *E. faecalis* (Bafna et al., 2020). Moreover, *E. faecalis* produces organic acids like acetic acid and lactic acid, and reactive oxygen species (ROS), particularly hydrogen peroxide, resulting in lowering pH and inhibiting sensitive bacteria like *E. coli* (Sajjad et al., 2024). The antimicrobial peptides (AMPs) produced by *E. faecalis* did not exhibit inhibitory activity against *S. aureus* and *B. cereus,* as evident from the absence of clear zones of inhibition around the wells in the agar plates. On the other hand, control wells with ciprofloxacin showed distinct inhibition zones in all plates, confirming the validity of the assay. These can be attributed to the thick peptidoglycan layers in the cell wall that prevent the entry of AMPs, efflux pumps (Sinha et al., 2024) that expel toxic compounds, and also due to the presence of proteolytic enzymes that degrade AMPs. *Enterococcus faecalis* is a primarily homofermentative lactic acid bacterium, and its antimicrobial activity is therefore likely dominated by lactic acid production along with other metabolic by-products rather than heterofermentative pathways.

### 3.9 Hemolysis and gelatinase activity

**Figure 11:**
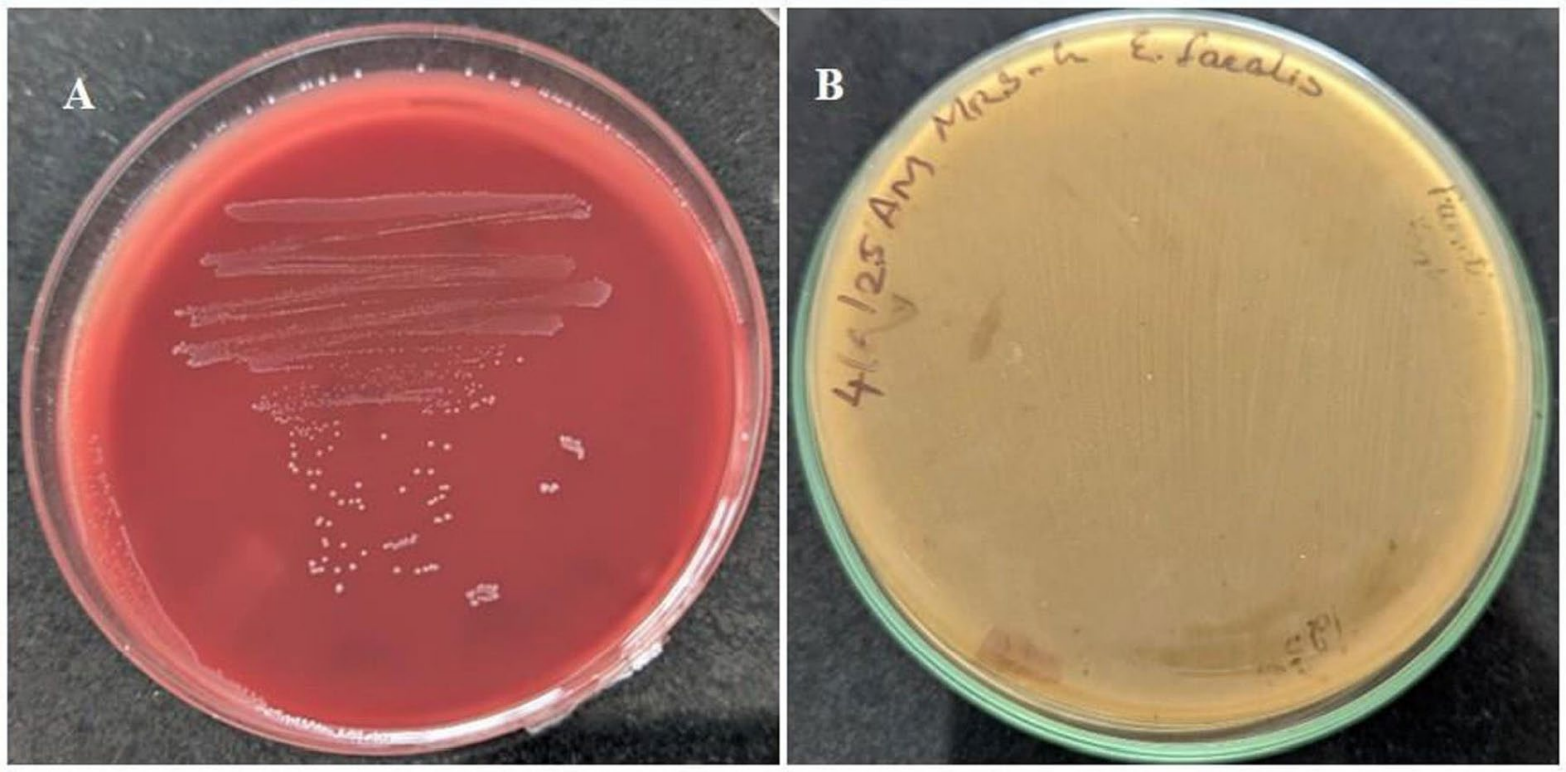
**(A)** *E. faecalis* RIT BM _S15-3_ on blood agar and **(B)** on MRS agar supplemented with 3% gelatin.

Given that *E. faecalis* strains are known to exhibit opportunistic pathogenicity, preliminary safety evaluation of *E. faecalis* RIT BM_S15-3_ was performed by assessing hemolytic and gelatinase activities. In this study, the isolate was found to be non-hemolytic (*ϒ*-hemolysis), as evidenced by the absence of a clear zone around the culture streak on blood agar (Figure 11A), indicating a lack of hemolysin production (Lad et al., 2022). Furthermore, the isolate tested negative for gelatinase activity, as no zone of hydrolysis was observed on gelatin agar (Figure 11B), consistent with the findings of Abdelrahman et al. (2024). The absence of hemolytic and gelatinase activities supports the non-pathogenic profile of the *E. faecalis* strain, reinforcing its suitability for probiotic applications. However, these assays represent only an initial safety screening, and the absence of hemolysis and gelatinase does not exclude the presence of other virulence determinants. Comprehensive safety evaluation through whole-genome sequencing is required for definitive safety assessment of additional virulence factors and confirmation at the genomic level.

### 3.10 Effect of substrate concentration on GABA production

**Figure 12:**
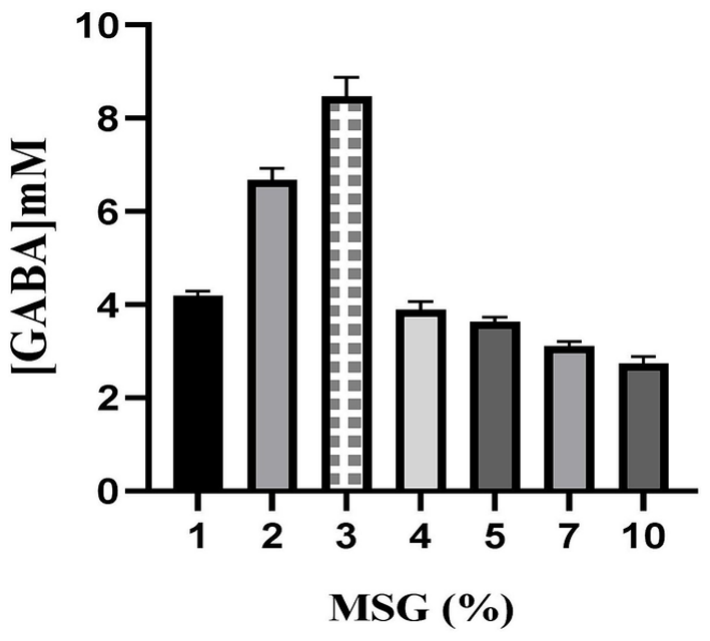
Effect of substrate concentration on the production of GABA. The given data represents the mean of three individual experiments, and error bars represent standard error (SD).

GABA production followed an increasing trend up to 3% of MSG concentration, and a slight decrease up to 5% MSG, steep decrease was found over 5% (Figure 12). The substrate concentration is optimal for glutamate decarboxylase (GAD) activity up to 3% MSG. This enhances GABA production due to increased substrate availability and efficient enzyme-substrate binding. Enzyme saturation or inhibition has occurred at this point. More than 3% of MSG significantly increased osmotic stress and cellular inhibition (Zhang et al., 2010). This resulted in a steep decline in GABA production (Tan et al., 2024). *E. faecalis* RIT BM_S15-3_ produced 8.4659 mM of GABA which is the maximum level produced among different concentrations of substrate. 3% of substrate was taken for the further experiments as an optimum substrate concentration. Availability of substrate as well as nutritional stress are imperative factors involved during GABA production. MRS with 3% MSG was determined as optimal condition yielding 8.46 mM GABA, while production lowered above 5% MSG due to osmotic stress as well as inhibition of enzymes. Similarly, optimum yields were at 37 °C as well as pH 6.8, agreeing with optimum GAD activity conditions (Xiong et al., 2017; Miao et al., 2024; Yee et al., 2024). These results confirm the potential of optimizing cultural as well as physiological condition for the scalability of GABA production. To address the relationship between microbial growth and GABA production with respect to substrate concentration, a 48 h growth profile along with corresponding GABA accumulation was evaluated (Supplementary Figures S1, S2). The results indicate that increased substrate availability enhanced GABA production up to an optimal level at 3% substrate concentration, where the microbial growth was at late stationary phase/decline phase. This reflects that microbial growth is not associated with GABA production.

### 3.11 Effect of temperature on GABA production

**Figure 13:**
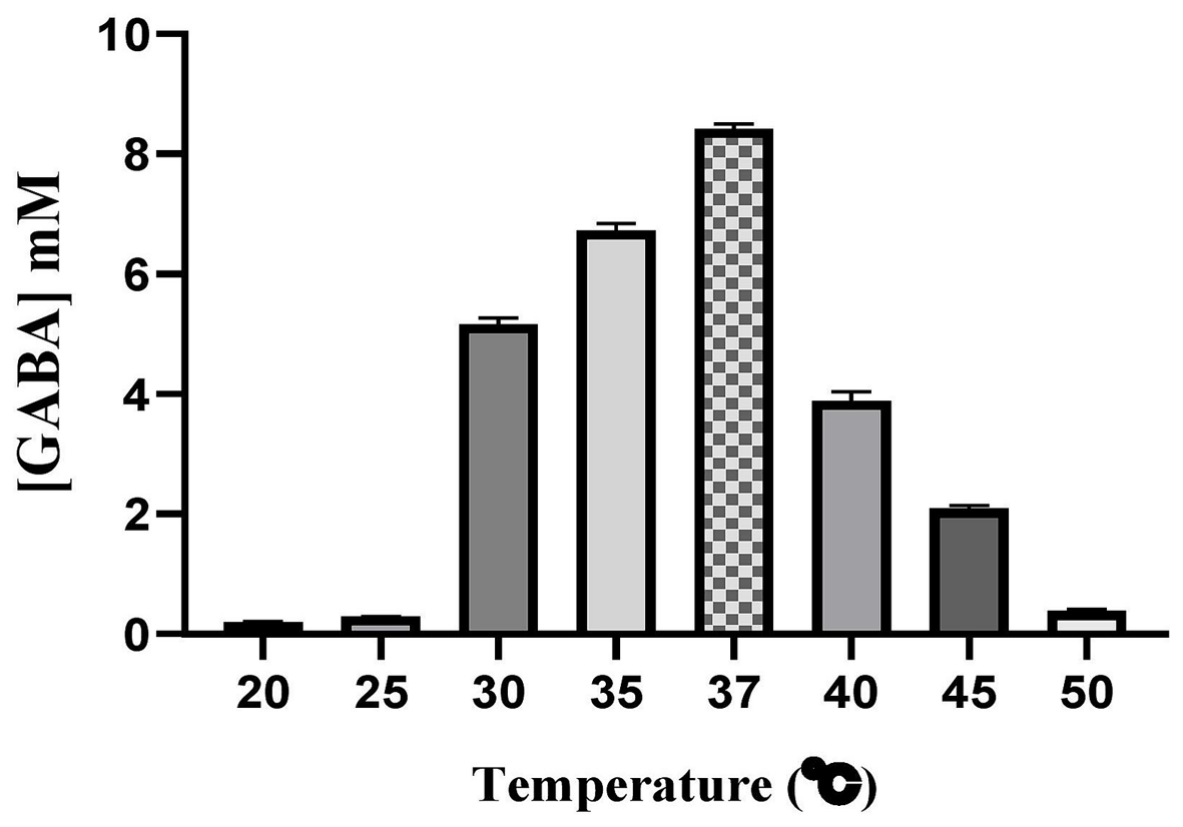
Effect of temperature on the production of GABA. The given data represents the mean of three separate experiments, and error bars represent standard error (SD).

As the temperature increases production of GABA also increased upto 37 °C and a steep decrease in GABA production was observed after 37 °C. Maximum production of 8.4242 mM was found at this temperature. At higher temperature, *E. faecalis* was unable to produce higher amount of GABA (Figure 13). *E. faecalis* RIT BM_S15-3_, being a mesophilic bacterium thrives best at moderate temperatures, typically between 30–37 °C. It is at 37 °C, that the cell’s metabolic processes, including synthesis of GABA using GAD are more active and has more stability. The GAD enzyme being highly sensitive to temperature likely maintains its optimal activity at 37 °C, which results in maximum GABA production. Rising of temperature beyond 37 °C can denature GAD enzyme reducing its catalytic efficiency. As the denatured or partially unfolded GAD is unable to maintain its active site, it leads to a steep decline in the synthesis of GABA (Xiong et al., 2017). The influence of temperature on growth and GABA production was assessed (Supplementary Figures S3, S4). Elevated temperatures (45 °C and 50 °C) resulted in reduced biomass formation, while GABA production was still detected, indicating that it is independent of active growth. Previous reports suggest that GABA production is stress-dependent rather than growth (Laroute et al., 2021).

### 3.12 Effect of pH on GABA production

**Figure 14:**
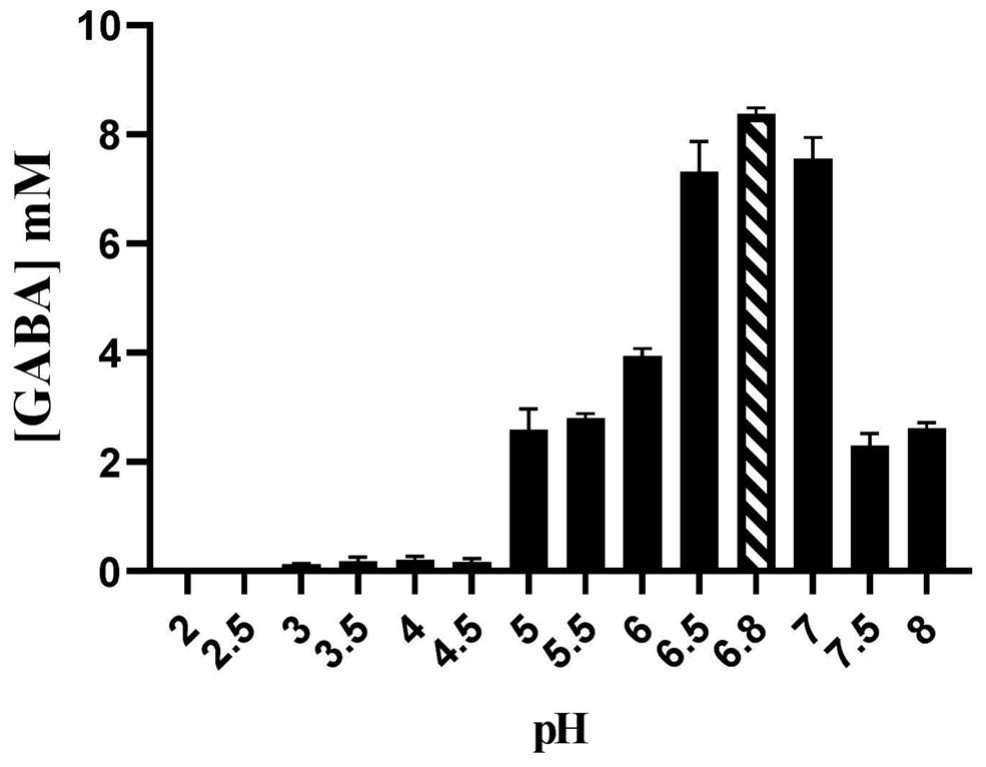
Effect of pH on the production of GABA. The given data represents the mean of three separate experiments.

The optimum pH for the production of GABA by *E. faecalis* RIT BM_S15-3_ was found to be 6.8 (Figure 14). The GABA produced was 8.37573 mM at optimum pH. The activity of the enzyme glutamate decarboxylase (GAD) is most efficient near neutral pH, supporting maximum GABA production (Miao et al., 2024). If the pH is maintained continuously near the optimal range (around pH 6.8) during fermentation, a higher and more sustained GABA yield can be expected. This is because stable pH conditions help maintain optimal glutamate decarboxylase (GAD) activity and prevent enzyme inhibition caused by excessive acidification during fermentation. In highly acidic and alkaline pH, a drastic decline has been observed, indicating enzyme inactivation or unfavorable reaction conditions. The effect of pH on both microbial growth and GABA production was evaluated further to check the role of microbial biomass in GABA production (Supplementary Figures S5, S6). Growth was markedly reduced under acidic conditions; however, GABA production remained detectable, suggesting activation of stress-responsive metabolic pathways.

In the present study, pH conditions were evaluated as initial set points; however, maintaining pH at the optimal level (pH 6.8) throughout fermentation could further enhance GABA yield by sustaining enzyme activity and metabolic flux. In controlled fermentation systems, strategies such as pH-stat regulation or buffering can be employed to prevent deviations from the optimal range, thereby improving production efficiency.

The comparative analysis of growth and metabolite production suggests that GABA synthesis by *E. faecalis* RIT BMS15-3 is not strictly growth-associated. The delayed accumulation of GABA, with maximum levels observed during the decline phase (Supplementary Figures S1, S3, S5), indicates its linkage to secondary metabolism and stress adaptation mechanisms.

Under conditions unfavorable for growth, such as acidic pH and elevated temperature, reduced biomass formation was observed; however, GABA production persisted (Supplementary Figures S2–S4). This supports the role of the glutamate decarboxylase system in acid stress response, where GABA synthesis contributes to intracellular pH homeostasis.

These findings highlight that GABA production may occur independently of active cell proliferation and is likely triggered as a protective metabolic response under environmental stress. The results also indicate the potential of *E. faecalis* RIT BM_S15-3_ to produce GABA and suggest its possible application in fermentation systems; however, further investigation is needed to analyze long-term production stability and industrial-scale feasibility.

In addition, process optimization approaches such as fed-batch fermentation with controlled substrate (MSG) feeding may help mitigate substrate inhibition and osmotic stress observed at higher concentrations. These strategies could enable sustained GABA production over extended periods and improve overall yield. Therefore, while the present study establishes the optimal conditions for GABA production, further investigation under controlled bioreactor conditions is required to evaluate process scalability and industrial applicability.

## 4 Conclusion

The current study emphasizes human breast milk as a promising and underexplored reservoir of psychobiotic microorganisms with GABA-producing potential. The identification of *Enterococcus faecalis* RIT BM_S15-3_ as a GABA-producing strain underscores its dual application as a probiotic candidate and as a functional culture for GABA enrichment in food systems. The observed tolerance to gastrointestinal stress conditions and its capacity for GABA production under physiologically relevant environments highlights its potential role in modulating the gut-brain axis. Moreover, the optimization of fermentation parameters support its feasibility for controlled GABA production, highlighting possible application in the development of GAB-enriched functional foods. However, given the opportunistic nature of *Enterococcus faecalis,* a complete safety evaluation, including assessment of virulence factors is necessary before application. Furthermore, *in vivo* studies are required to validate its psychobiotic efficacy and functional impact. Future work should also focus on scale-up of the process to enable its industrial application.

In summary, this study provides a foundation for exploring breast milk-derived psychobiotics while emphasizing the need for rigorous validation to ensure safe and effective application.

## Data Availability

The datasets generated for this study can be found in the NCBI GenBank repository under accession number PZ055568.

## References

[ref1] Abdelrahman K. A. Kashef M. T. Aziz R. K. Hashem Y. A. (2024). Genotype–phenotype correlation of fecal Streptococcus regulator (fsr) locus with gelatinase activity and biofilm formation intensity in clinical *E. faecalis* isolates. Future J. Pharm. Sci. 10:37. doi: 10.1186/s43094-024-00610-8

[ref3] Al Atya A. K. Drider-Hadiouche K. Ravallec R. Silvain A. Vachee A. Drider D. (2015). Probiotic potential of *Enterococcus faecalis* strains isolated from meconium. Front. Microbiol. 6:227. doi: 10.3389/fmicb.2015.00227, 25883590 PMC4382979

[ref4] Alang H. Kusnadi J. Ardyati T. (2020). Optimization and characterization of enterocin *Enterococcus faecalis* K2B1 isolated from Toraja’s belang buffalo milk, South Sulawesi, Indonesia. Biodiversitas J. Biol. Divers. 21, 1236–1242. doi: 10.13057/biodiv/d210351

[ref5] Alkay Z. Şensoy A. T. Durak M. Z. Tuncil Y. E. Dertli E. (2025). Identification of GABA producing strains from sourdoughs and optimization of GABA production by response surface-integrated particle swarm approach. Prep. Biochem. Biotechnol. 55, 1221–1230. doi: 10.1080/10826068.2025.2498456, 40306325

[ref6] Anifowose O. R. Adeoye B. O. Olayiwola A. O. (2024). Pathogenicity and antibiotic susceptibility pattern of *Enterococcus faecalis* in *Clarias gariepinus* juvenile. Int. J. Oceanogr. Aquac. 8:000303. doi: 10.23880/ijoac-16000303

[ref7] Augustine E. I. (2024). Identification of *Proteus Vulgaris* using series of biochemical test and staining technique: ecology, pathogenicity (enteric pathogen), description and prevention of it’s diseases. Int. J. Contemp. Microbiol. 10. doi: 10.37506/c4fpes96

[ref8] Ayyash M. Liu S. Q. (2023). Special issue “probiotics, prebiotics and functional foods: health benefits and biosafety”. Microorganisms 11:1218. doi: 10.3390/microorganisms11051218, 37317192 PMC10222182

[ref9] Bafna J. A. Sans-Serramitjana E. Acosta-Gutiérrez S. Bodrenko I. V. Hörömpöli D. Berscheid A. . (2020). Kanamycin uptake into *Escherichia coli* is facilitated by OmpF and OmpC porin channels located in the outer membrane. ACS Infect. Dis. 6, 1855–1865. doi: 10.1021/acsinfecdis.0c00102, 32369342

[ref10] Bakshi I. Dey S. Raut A. J. Katta S. Sharma P. (2024). Exploring the gut-brain axis: a comprehensive review of interactions between the gut microbiota and the central nervous system. Int. J. Multidiscipl. Res. 6:1. doi: 10.36948/ijfmr.2024.v06i03.19563

[ref11] Benarroch E. (2021). What is the role of GABA transporters in seizures? Neurology 97, 580–584. doi: 10.1212/WNL.0000000000012574

[ref12] Bock H. J. Lee N. K. Paik H. D. (2023). Neuroprotective effects of heat-killed Levilactobacillus brevis KU15152 on H2O2-induced oxidative stress. J. Microbiol. Biotechnol. 33, 1189–1196. doi: 10.4014/jmb.2304.04045, 37317628 PMC10580890

[ref13] Braga J. D. Thongngam M. Kumrungsee T. (2024). Gamma-aminobutyric acid as a potential postbiotic mediator in the gut–brain axis. npj Sci. Food 8:16. doi: 10.1038/s41538-024-00253-2, 38565567 PMC10987602

[ref14] Casertano M. Fryganas C. Valentino V. Troise A. D. Vitaglione P. Fogliano V. . (2024). Gut production of GABA by a probiotic formula: an *in vitro* study. Benefic. Microbes 15, 67–81. doi: 10.1163/18762891-20230025, 38350463

[ref15] Chen D. Chen C. Guo C. Zhang H. Liang Y. Cheng Y. . (2023). The regulation of simulated artificial oro-gastrointestinal transit stress on the adhesion of *Lactobacillus plantarum* S7. Microb. Cell Factories 22:170. doi: 10.1186/s12934-023-02174-3, 37660047 PMC10474686

[ref16] Chen Z. Leng X. Zhou F. Shen W. Zhang H. Yu Q. . (2023). Screening and identification of probiotic lactobacilli from the infant gut microbiota to alleviate lead toxicity. Probiotics Antimicrobial Proteins 15, 821–831. doi: 10.1007/s12602-021-09895-0, 35060081

[ref17] Cho Y. R. Chang J. Y. Chang H. C. (2007). Production of gamma-aminobutyric acid (GABA) by *Lactobacillus buchneri* isolated from kimchi and its neuroprotective effect on neuronal cells. J. Microbiol. Biotechnol. 17, 104–109. 18051360

[ref18] Choi G. H. Bock H. J. Lee N. K. Paik H. D. (2022). Soy yogurt using lactobacillus plantarum 200655 and fructooligosaccharides: neuroprotective effects against oxidative stress. J. Food Sci. Technol. 59, 4870–4879. doi: 10.1007/s13197-022-05575-1, 36276546 PMC9579260

[ref20] Christodoulou G. N. (2020). Mental health promotion: person-centered perspective. Int. J. Person Centered Med. 10, 53–68. doi: 10.5750/ijpcm.v10i1.1049

[ref21] Comerlato C. B. Prichula J. Siqueira F. M. Ritter A. C. Varela A. P. M. Mayer F. Q. . (2022). Genomic analysis of *Enterococcus durans* LAB18S, a potential probiotic strain isolated from cheese. Genet. Mol. Biol. 45:e20210201. doi: 10.1590/1678-4685-gmb-2021-0201, 35244137 PMC8894896

[ref22] Cui J. Wang W. Tang Y. Feng S. Liu H. Hao Z. (2025). Application of psychobiotics in clinical treatment of mental disorders: neurodevelopmental disorders, neurodegenerative diseases, depression and anxiety. Interdiscip. Med. 3:e20240041. doi: 10.1002/INMD.20240041

[ref23] de la Ochoa- Paz L. D. Gulias-Cañizo R. Ruíz-Leyja E. D. Sánchez-Castillo H. Parodí J. (2021). The role of GABA neurotransmitter in the human central nervous system, physiology, and pathophysiology. Rev. Mex. Neuroci. 22, 67–76. doi: 10.24875/rmn.20000050

[ref24] Debnath A. Miyoshi S. I. (2024). Effect of physicochemical and microbiological factors on the development of viable but non-culturable and resuscitation states of *Vibrio cholerae*. Arch. Microbiol. 206:224. doi: 10.1007/s00203-024-03956-y, 38642319

[ref25] Denysov Y. Putyatin G. Moroz S. Semenikhina V. (2023). Effects of probiotic supplement *Lactobacillus Plantarum* CECT7485 and *Lactobacillus Brevis* CECT7480 on sleep quality in patients with anxiety and depression comorbidity. Eur. Psychiatry 66, S454–S454. doi: 10.1192/j.eurpsy.2023.976

[ref26] Elias H. Rosenblum D. (2024). Selective Serotonin Reuptake Inhibitors (SSRIs). New York: Oxford University Press, 397–399.

[ref27] Eskandari M. Faraz S. M. Hosseini S. E. Moradi S. Saeidian H. (2023). Electron ionization mass spectrometry fragmentation routes of chemical weapons convention-related organoarsenic compounds: Electron ionization and density functional theory studies. Rapid Commun. Mass Spectrom. 37:e9511. doi: 10.1002/rcm.9511, 36945901

[ref28] Faizullina R. A. Ukraintsev S. E. (2024). The role of *Lactobacillus reuteri* in health formation. Vopr. Detskoj Dietol. 22, 38–44. doi: 10.20953/1727-5784-2024-4-38-44

[ref29] Ferreira S. M. Hagan D. W. Phelps E. (2022). 1470-p: GABA stimulates or inhibits insulin and somatostatin secretion in human pancreatic islets depending on glucose concentration. Diabetes 71:1470-P. doi: 10.2337/db22-1470-P

[ref30] Gangaraju D. Murty V. R. Prapulla S. G. (2014). Probiotic-mediated biotransformation of monosodium glutamate to γ-aminobutyric acid: differential production in complex and minimal media and kinetic modelling. Ann. Microbiol. 64, 229–237. doi: 10.1007/s13213-013-0655-4

[ref31] Gaur P. Hada V. Rath R. S. Mohanty A. Singh P. Rukadikar A. (2023). Interpretation of antimicrobial susceptibility testing using European committee on antimicrobial susceptibility testing (EUCAST) and clinical and laboratory standards institute (CLSI) breakpoints: analysis of agreement. Cureus 15:e36977. doi: 10.7759/cureus.36977, 37139290 PMC10149341

[ref32] Ghith A. Maleki R. Grzeskowiak L. E. Amir L. H. Ingman W. V. (2025). Challenges and opportunities in quantifying bioactive compounds in human breastmilk. Biomolecules 15:325. doi: 10.3390/biom15030325, 40149861 PMC11940641

[ref33] Gill P. K. (2023). Lactic acid bacteria as probiotics: current status and future prospects. Asian J. Microbiol. Biotechnol. Environ. Sci. 8, 120–139. doi: 10.56557/AJMAB/2023/v8i28424

[ref34] Greenwood M. Murciano-Martinez P. Berrington J. Flitsch S. L. Austin S. Stewart C. (2024). Characterising glycosaminoglycans in human breastmilk and their potential role in infant health. Microbial Cell 11, 221–234. doi: 10.15698/mic2024.07.827, 38975022 PMC11224681

[ref35] Gupta S. Sundar S. K. (2024). Kinetics of microbial growth, substrate consumption, and product formation. In: Dhagat, S., Jujjavarapu, S.E., Sampath Kumar, N., Mahapatra, C. (eds), Recent Advances in Bioprocess Engineering and Bioreactor Design. Singapore: Springer. doi: 10.1007/978-981-97-1451-3_5

[ref36] Haddad G. Takakura T. Bellali S. Fontanini A. Ominami Y. Khalil J. B. . (2022). A preliminary investigation into bacterial viability using scanning electron microscopy–energy-dispersive X-ray analysis: the case of antibiotics. Front. Microbiol. 13:967904. doi: 10.3389/fmicb.2022.967904, 36003945 PMC9393632

[ref37] Hagan D. W. Ferreira S. M. Santos G. J. Phelps E. A. (2022). The role of GABA in islet function. Front. Endocrinol. 13:972115. doi: 10.3389/fendo.2022.972115, 36246925 PMC9558271

[ref38] Hamed E. (2021). Isolation, characterization and identification of lactic acid bacteria as probiotic. Ann. Agric. Sci. Moshtohor 59, 311–322. doi: 10.21608/assjm.2021.194809

[ref39] Heath K. E. Feduska J. M. Taylor J. P. Houp J. A. Botta D. Lund F. E. . (2023). GABA and combined GABA with GAD65-alum treatment alters Th1 cytokine responses of PBMCs from children with recent-onset type 1 diabetes. Biomedicine 11:1948. doi: 10.3390/biomedicines11071948, 37509587 PMC10377053

[ref40] Holt J. G. Krieg N. R. Sneath P. H. A. Staley J. T. Williams S. T. (1994). Bergey's Manual of Determinate Bacteriology. Available online at: https://agris.fao.org/search/en/providers/122621/records/647396ad68b4c299a3fb6447 (Accessed April 1, 2026).

[ref41] Hui W. Sun L. Zhang H. Zou L. Zou Q. Ouyang P. (2016). Quantitative analysis of ripasudil hydrochloride hydrate and its impurities by reversed-phase high-performance liquid chromatography after precolumn derivatization: identification of four impurities. J. Sep. Sci. 39, 3302–3310. doi: 10.1002/jssc.201600278, 27390135

[ref42] Ibrahim N. F. Aris F. Mohd Jalil M. T. Mohamed Yunus N. Ab Rashid S. Zakaria N. A. (2024). Application of lactic acid bacteria-derived GABA in food industry: GABA-producing strains, biosynthesis, and health benefits. Int. Food Res. J. 31, 1076–1093. doi: 10.47836/ifrj.31.5.01

[ref43] Ibrahim A. S. Bayomy R. H. Hussein R. A. Yousef U. M. Elazab W. E. (2022). Suicidal ideation and behavior among subjects with substance abuse disorder related to pregabalin. Middle East Curr. Psychiatry 29:37. doi: 10.1186/s43045-022-00205-0

[ref44] Imran A. Faisal F. Jameel A. Aslam R. Jabbar I. (2025). Exploring the role of psychobiotics in mental and emotional well-being and how psychobiotics can help fight anxiety and depression. Indus J. Biosci. Res. 3, 51–56. doi: 10.70749/ijbr.v3i3.823

[ref45] Islam M. D. Harrison B. D. Li J. J. Y. McLoughlin A. G. Court D. A. (2023). Do mitochondria use efflux pumps to protect their ribosomes from antibiotics? Microbiology 169:001272. doi: 10.1099/mic.0.001272, 36748523 PMC9993110

[ref46] Kapreliants L. Zhuk E. (2021). PSYCHOBIOTICS–probiotics that elevate mood. Food Sci. Technol. 15, 4–14. doi: 10.15673/fst.v15i1.1969

[ref47] Karim R. (2023). Human milk oligosaccharides (HMOS) for infant health and microbiome development. World Nutr. J. 7, 16–16. doi: 10.25220/v07.s1.0015

[ref48] Khushboo Karnwal A. Malik T. (2023). Characterization and selection of probiotic lactic acid bacteria from different dietary sources for development of functional foods. Front. Microbiol. 14:1170725. doi: 10.3389/fmicb.2023.1170725 37213505 PMC10196247

[ref49] Kim G. H. Baek K. R. Lee G. E. Lee J. H. Moon J. H. Seo S. O. (2025). Development of starter cultures for precision fermentation of kombucha with enriched gamma-aminobutyric acid (GABA) content. Fermentation 11:17. doi: 10.3390/fermentation11010017

[ref50] Kim Y. Choi S. I. Jeong Y. Kang C. H. (2022). Evaluation of safety and probiotic potential of *Enterococcus faecalis* MG5206 and *Enterococcus faecium* MG5232 isolated from kimchi, a Korean fermented cabbage. Microorganisms 10:2070. doi: 10.3390/microorganisms10102070, 36296346 PMC9607435

[ref51] Kim M. A. Rosa V. Min K. S. (2020). Characterization of *Enterococcus faecalis* in different culture conditions. Sci. Rep. 10:21867. doi: 10.1038/s41598-020-78998-5, 33318537 PMC7736865

[ref52] Kyei-Baffour V. O. Vijaya A. K. Burokas A. Daliri E. B. M. (2025). Psychobiotics and the gut-brain axis: advances in metabolite quantification and their implications for mental health. Crit. Rev. Food Sci. Nutr. 65, 7085–7104. doi: 10.1080/10408398.2025.2459341, 39907087

[ref53] Lad V. Panchal D. Pithawala M. Dwivedi M. K. Amaresan N. (2022). Determination of hemolytic activity. In: Dwivedi, M. K., Amaresan, N., Sankaranarayanan, A., Begum, R. (eds), Biosafety assessment of probiotic potential. Methods and Protocols in Food Science. New York, NY: Humana. doi: 10.1007/978-1-0716-2509-5_6

[ref54] Laroute V. Mazzoli R. Loubière P. Pessione E. Cocaign-Bousquet M. (2021). Environmental conditions affecting GABA production in *Lactococcus lactis* NCDO 2118. Microorganisms 9:122. doi: 10.3390/microorganisms9010122, 33430203 PMC7825684

[ref55] Lee J. S. Kim K. S. (2023). Optimization of culture conditions for and assessment of kimchi-originated lactic acid bacterial isolates toward their extracellular GABA-producing ability. Emir. J. Food Agric. 35, 1033–1040 Available online at: http://www.ejfa.me/

[ref56] Li H. Gao D. Cao Y. Xu H. (2008). A high γ-aminobutyric acid-producing *Lactobacillus brevis* isolated from Chinese traditional paocai. Ann. Microbiol. 58, 649–653. doi: 10.1007/BF03175570

[ref57] Lima-Silveira L. Hasser E. M. Kline D. D. (2022). Cardiovascular deconditioning increases GABA signaling in the nucleus tractus solitarii. J. Neurophysiol. 128, 28–39. doi: 10.1152/jn.00102.2022, 35642806 PMC9236861

[ref58] Liu Z. Mu C. Zhu W. (2022). IDDF2022-ABS-0050 Gaba-producing potential of GUT microbiota and the response to antibiotic intervention. Gut 71, A38–A38. doi: 10.1136/gutjnl-2022-iddf.39

[ref59] Liu W. Zhang Y. Chen J. Li X. Huang Y. Zhao F. . (2025). Global burden and trends of major mental disorders in individuals under 24 years of age from 1990 to 2021, with projections to 2050: insights from the global burden of disease study 2021. Front. Public Health 13:1635801. doi: 10.3389/fpubh.2025.1635801, 41036122 PMC12481897

[ref60] Lopez K. R. Traña P. S. Mayorga R. Z. Alpízar Y. R. Vellanero M. J. A. Obando M. G. G. (2025). Probióticos y prebióticos en la prevención y tratamiento de enfermedades gastrointestinales [Probiotics and prebiotics in the prevention and treatment of gastrointestinal diseases]. Revista Médica PortalesMedicos.com, Article 221.

[ref61] Lu X. Xie C. H. Gu Z. (2009). Optimisation of fermentative parameters for GABA enrichment by *Lactococcus lactis*. Czech J. Food Sci. 27, 433–442. doi: 10.17221/45/2009-CJFS

[ref62] Luo H. Liu Z. Xie F. Bilal M. Liu L. Yang R. . (2021). Microbial production of gamma-aminobutyric acid: applications, state-of-the-art achievements, and future perspectives. Crit. Rev. Biotechnol. 41, 491–512. doi: 10.1080/07388551.2020.1869688, 33541153

[ref64] Meyers S. Tamir M. (2024). Emotion regulation versus mood regulation. Emot. Rev. 16, 151–161. doi: 10.1177/17540739241259559

[ref65] Miao L. Zheng Y. Cheng R. Liu J. Zheng Z. Yang H. . (2024). Efficient synthesis of γ-aminobutyric acid from monosodium glutamate using an engineered glutamate decarboxylase active at a neutral pH. Catalysts 14:905. doi: 10.3390/catal14120905

[ref66] Michlig N. Amirav A. Neumark B. Lehotay S. J. (2024). Comparison of different fast gas chromatography–mass spectrometry techniques (cold EI, MS/MS, and HRMS) for the analysis of pyrethroid insecticide residues in food. Anal. Methods 16, 5599–5618. doi: 10.1039/d4ay00858h, 39101707

[ref67] Mick G. J. McCormick K. L. (2024). The role of GABA in type 1 diabetes. Front. Endocrinol. 15:1453396. doi: 10.3389/fendo.2024.1453396, 39619323 PMC11604429

[ref68] Monteiro M. P. Kohl H. M. Roullet J. B. Gibson K. M. Ochoa-Repáraz J. Castillo A. R. (2024). Genetically engineered *Lactococcus lactis* strain constitutively expresses GABA-producing genes and produces high levels of GABA. Lett. Appl. Microbiol. 77:ovae051. doi: 10.1093/lambio/ovae051, 38816215 PMC11187484

[ref69] Munoz F. Gao Q. Mattanovich M. Trost K. Hodek O. Lindqvist A. . (2025). Lysine potentiates insulin secretion via AASS-dependent catabolism and regulation of GABA content and signaling. bioRxiv. doi: 10.1101/2025.03.03.641187, 41167553

[ref70] Nwankwo A. Koyyalagunta D. Huh B. D'Souza R. S. Javed S. (2024). A comprehensive review of the typical and atypical side effects of gabapentin. Pain Pract. 24, 1051–1058. doi: 10.1111/papr.13400, 38949515

[ref71] Oleskin A. V. Shenderov B. A. (2019). Probiotics and psychobiotics: the role of microbial neurochemicals. Probiotics Antimicrobial Proteins 11, 1071–1085. doi: 10.1007/s12602-019-09583-0, 31493127

[ref72] Oyovwi M. O. Udi O. A. (2025). The gut-brain axis and neuroinflammation in traumatic brain injury. Mol. Neurobiol. 62, 4576–4590. doi: 10.1007/s12035-024-04585-8, 39466574

[ref73] Panigrahi P. (2024). The neonatal gut microbiome and global health. Gut Microbes 16:2308965. doi: 10.1080/19490976.2024.2352175, 38743045 PMC11095572

[ref75] Patsalos P. N. (2022). Antiseizure Medication Interactions: A Clinical guide. Cham: Springer Nature.

[ref76] Pressey J. C. de Saint-Rome M. Raveendran V. A. Woodin M. A. (2023). Chloride transporters controlling neuronal excitability. Physiol. Rev. 103, 1095–1135. doi: 10.1152/physrev.00025.2021, 36302178

[ref77] Rashid H. Anwar H. Baig F. M. Mukhtar I. Muhammad T. Zaidi A. (2025). Potentially probiotic NPL 1334 strain of *Enterococcus durans* benefits rats with diet-induced hypercholesterolemia. BMC Biotechnol. 25:7. doi: 10.1186/s12896-024-00943-5, 39825321 PMC11740586

[ref78] Rashid G. Sharma L. Khan N. (2024). Probiotics: a Comprehensive guide to Enhance Health and Mitigate Disease. 1st Edn Boca Raton: CRC Press.

[ref9001] Ratanaburee A. Kantachote D. Charernjiratrakul W. Penjamras P. Chaiyasut C. (2011). Enhancement of γ-aminobutyric acid in a fermented red seaweed beverage by starter culture Lactobacillus plantarum DW12. Electron. J. Biotechnol, 14, 1–1. doi: 10.2225/vol14-issue3-fulltext-2

[ref79] Reiter M. A. Bradley T. Büchel L. A. Keller P. Hegedis E. Gassler T. . (2024). A synthetic methylotrophic *Escherichia coli* MTCC 42 as a chassis for bioproduction from methanol. Nature Catalysis 7, 560–573. doi: 10.1038/s41929-024-01137-0, 38828428 PMC11136667

[ref80] Sajjad Z. Sajjad F. Khan M. F. Manzoor S. Abubakar M. (2024). Efficacy of lactic acid and acetic acid against multidrug resistant *Staphylococcus aureus*, *E. Coli* and *Klebsiella* sp. Int. J. Vet. Sci. Res. 9, 11–19. doi: 10.18488/ijvsr.v9i2.4021

[ref81] Shabel S. J. Proulx C. D. Piriz J. Malinow R. (2014). GABA/glutamate co-release controls habenula output and is modified by antidepressant treatment. Science 345, 1494–1498. doi: 10.1126/science.1250469, 25237099 PMC4305433

[ref82] Sharma P. Singh N. Singh S. Khare S. K. Nain P. K. S. Nain L. (2022). Potent γ-amino butyric acid producing psychobiotic *Lactococcus lactis* LP-68 from non-rhizospheric soil of *Syzygium cumini* (black plum). Arch. Microbiol. 204:82. doi: 10.1007/s00203-021-02629-4, 34958412

[ref83] Shi Y. Zhai M. Li J. Li B. (2020). Evaluation of safety and probiotic properties of a strain of *Enterococcus faecium* isolated from chicken bile. J. Food Sci. Technol. 57, 578–587. doi: 10.1007/s13197-019-04089-7, 32116367 PMC7016052

[ref84] Singh P. Zhawar V. K. (2024). First report on GABA (gamma-aminobutyric acid) regulation of carbohydrates under wheat stripe rust in India. Arch. Phytopathol. Plant Protect. 57, 54–74. doi: 10.1080/03235408.2024.2336897

[ref85] Sinha S. Aggarwal S. Singh D. V. (2024). Efflux pumps: gatekeepers of antibiotic resistance in *Staphylococcus aureus* biofilms. Microbial Cell 11, 368–377. doi: 10.15698/mic2024.11.839, 39568862 PMC11576857

[ref86] Somekawa N. Sekine T. Hamada-Sato N. (2024). Inhibition of blood pressure elevation in GABA-enriched Hidakakombu fermented with *Lactiplantibacillus plantarum* 002 protected addition of trehalose. Int. J. Food Sci. Technol. 59, 1572–1579. doi: 10.1111/ijfs.16906

[ref88] Suharsono H. Suardana I. W. Pinatih K. J. P. (2023). Bacteriocin potency test of lactic acid bacteria (LAB) isolated from rumen of Bali cattle against low pH and bile salt. Bali Med. J. 12, 1142–1146. doi: 10.15562/bmj.v12i1.4305

[ref89] Sumalata G. (2023). Isolation and identification of indigenous probiotics from dairy products and human breast milk. J. Adv. Sci. Res. 14, 40–49. doi: 10.55218/JASR.202314806

[ref90] Sun T. Song B. Li B. (2025). Gut microbiota and atrial cardiomyopathy. Front. Cardiovasc. Med. 12:1210453. doi: 10.3389/fcvm.2025.1541278, 39968343 PMC11832500

[ref91] Takagi H. Kozuka K. Mimura K. Nakano S. Ito S. (2022). Front cover: design of a full-consensus glutamate decarboxylase and its application to GABA biosynthesis (ChemBioChem 8/2022). Chembiochem 23:e202100586. doi: 10.1002/cbic.202100586 34545992

[ref92] Tan S. W. Zu Koh Y. Siva S. Wasoh H. Mohamed M. S. Sobri Z. M. . (2024). Optimization of fermentative parameters to improve gamma-aminobutyric acid (GABA) production by *Lactiplantibacillus plantarum* B13. J. Biochem. Microbiol. Biotechnol. 12, 7–16. doi: 10.54987/jobimb.v12i1.935

[ref94] Taye Y. Degu T. Fesseha H. Mathewos M. (2021). Isolation and identification of lactic acid bacteria from cow milk and milk products. Sci. World J. 2021:4697445. doi: 10.1155/2021/4697445, 34421398 PMC8371643

[ref95] Toupal S. Coşansu S. (2024). Effects of freeze-dried banana and watermelon peel powders on bile salt resistance, growth kinetics, and survival of probiotic bacteria. Probiotics Antimicrobial Proteins 16, 1762–1772. doi: 10.1007/s12602-023-10131-0, 37535210

[ref96] Vaccalluzzo A. Agolino G. Pino A. Cristofolini M. Tagliazucchi D. Cattivelli A. . (2025). Gastrointestinal survivability of a BSH-positive Lacticaseibacillus rhamnosus VB4 strain and its effect on bile acid Deconjugation in a dynamic *in vitro* gut model. Nutrients 17:3179. doi: 10.3390/nu17193179, 41097256 PMC12526281

[ref97] Vitellio P. Chira A. De Angelis M. Dumitrascu D. L. Portincasa P. (2020). Probiotics in psychosocial stress and anxiety. A systematic review. J. Gastrointestin. Liver Dis. 29, 77–83. doi: 10.15403/jgld-352, 32176751

[ref98] Xiong Q. Xu Z. Xu L. Yao Z. Li S. Xu H. (2017). Efficient production of γ-GABA using recombinant *E. coli* expressing glutamate decarboxylase (GAD) derived from eukaryote *Saccharomyces cerevisiae*. Appl. Biochem. Biotechnol. 183, 1390–1400. doi: 10.1007/s12010-017-2506-4, 28656550

[ref99] Yao C. Shi F. Wang X. (2023). Chromosomal editing of *Corynebacterium glutamicum* ATCC 13032 to produce gamma-aminobutyric acid. Biotechnol. Appl. Biochem. 70, 7–21. doi: 10.1002/bab.2324, 35106837

[ref100] Yee C. S. Ilham Z. Cheng A. Abd Rahim M. H. Hajar-Azhari S. Yuswan M. H. . (2024). Optimisation of fermentation conditions for the production of gamma-aminobutyric acid (GABA)-rich soy sauce. Heliyon 10:e33147. doi: 10.1016/j.heliyon.2024.e33147 39040394 PMC11261068

[ref101] Yehuala G. A. Choe J. Shibeshi N. T. Delessa K. Desalegn A. Park M. K. (2025). Lactic acid bacteria from Ethiopian traditional beverage, Tella: technological and metabolic profiles for industrial application. J. Microbiol. 63:e2409008. doi: 10.71150/jm.2409008, 39895073 PMC13577074

[ref102] Zhang Y. Gao N.F. Feng Y. Song L. Gao Q. (2010). Biotransformation of sodium L-glutamate to gamma-aminobutyric acid by *L. brevis* TCCC13007 with two glutamate decarboxylase genes. In 2010 4th International Conference on Bioinformatics and Biomedical Engineering (pp. 1–4).

[ref103] Zhang Z. Liang F. Yan S. Chen Y. Guo B. Chen B. . (2025). Detection of aristolochic acids based on chemical derivatization and GC–MS/MS. J. Iran. Chem. Soc. 22, 903–914. doi: 10.1007/s13738-025-03197-w

