# Supplementary figures and images for: In vitro identification of GABA-producing psychobiotic candidates from human breast milk
Source: Front Microbiol. 2026 Apr 15;17:1715064. doi: 10.3389/fmicb.2026.1715064 (PMC13125111; doi:10.3389/fmicb.2026.1715064)

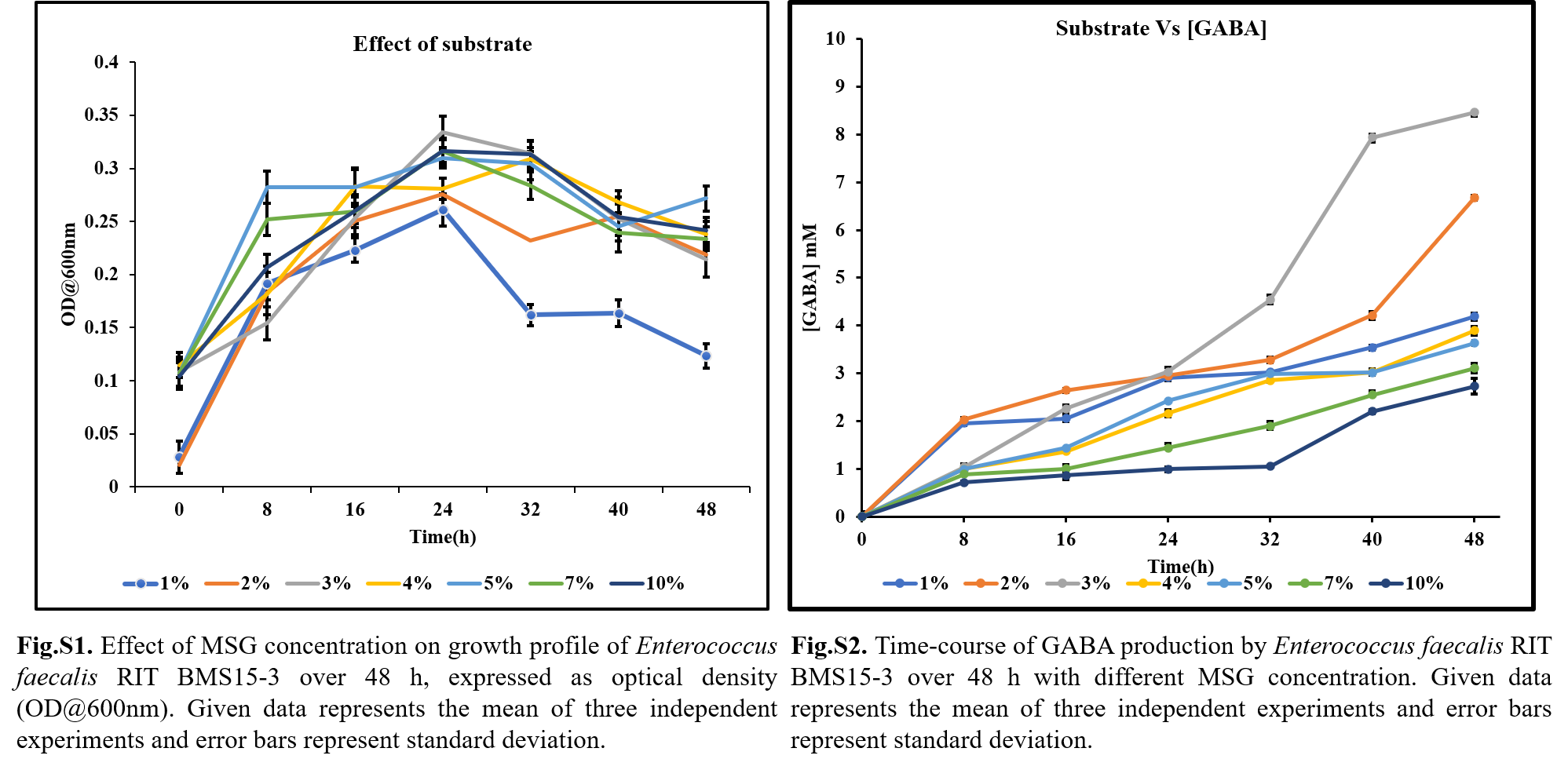

Supplement: Supplementary file 1 [file Image_1.TIF]

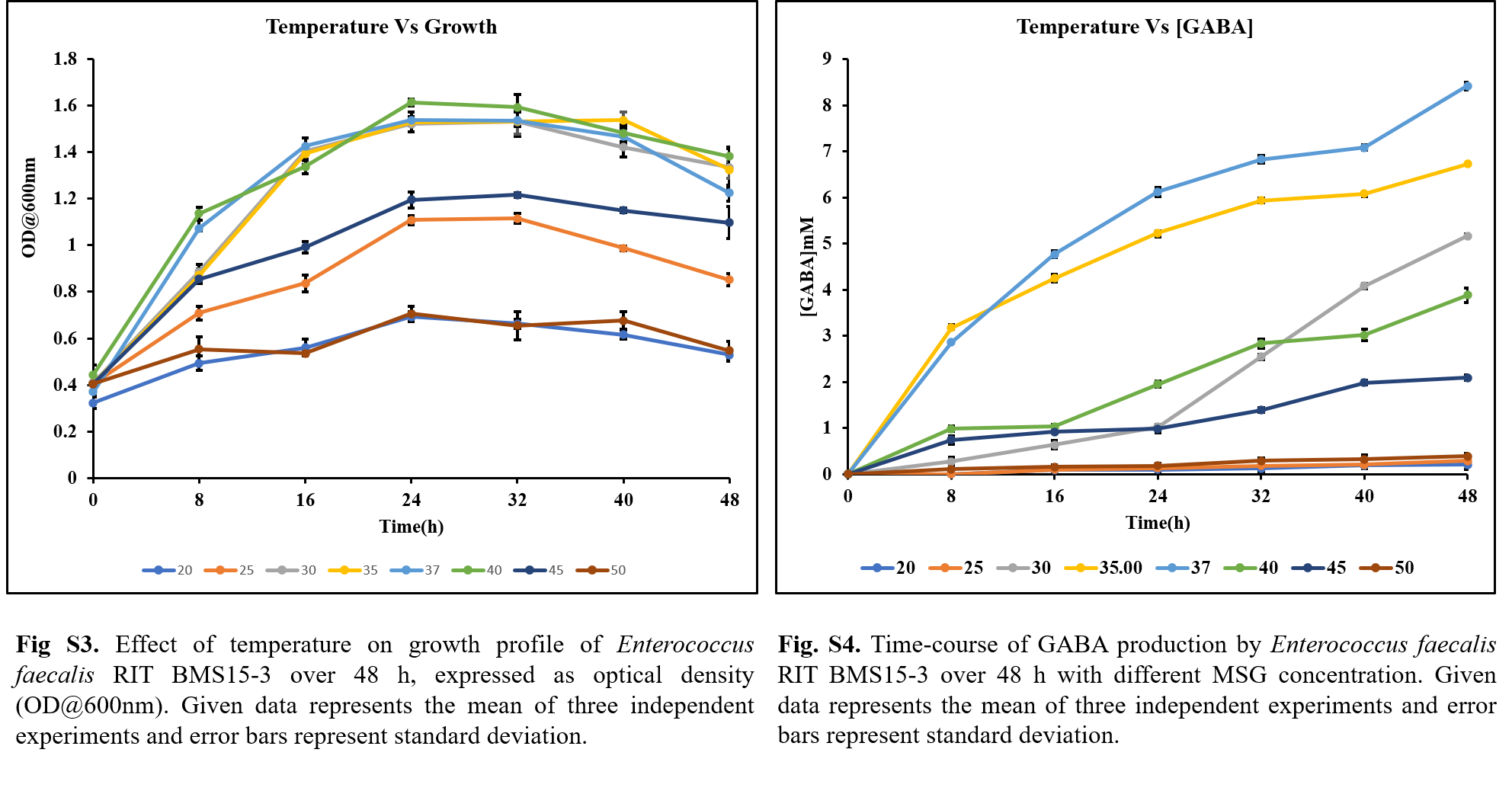

Supplement: Supplementary file 2 [file Image_2.TIF]

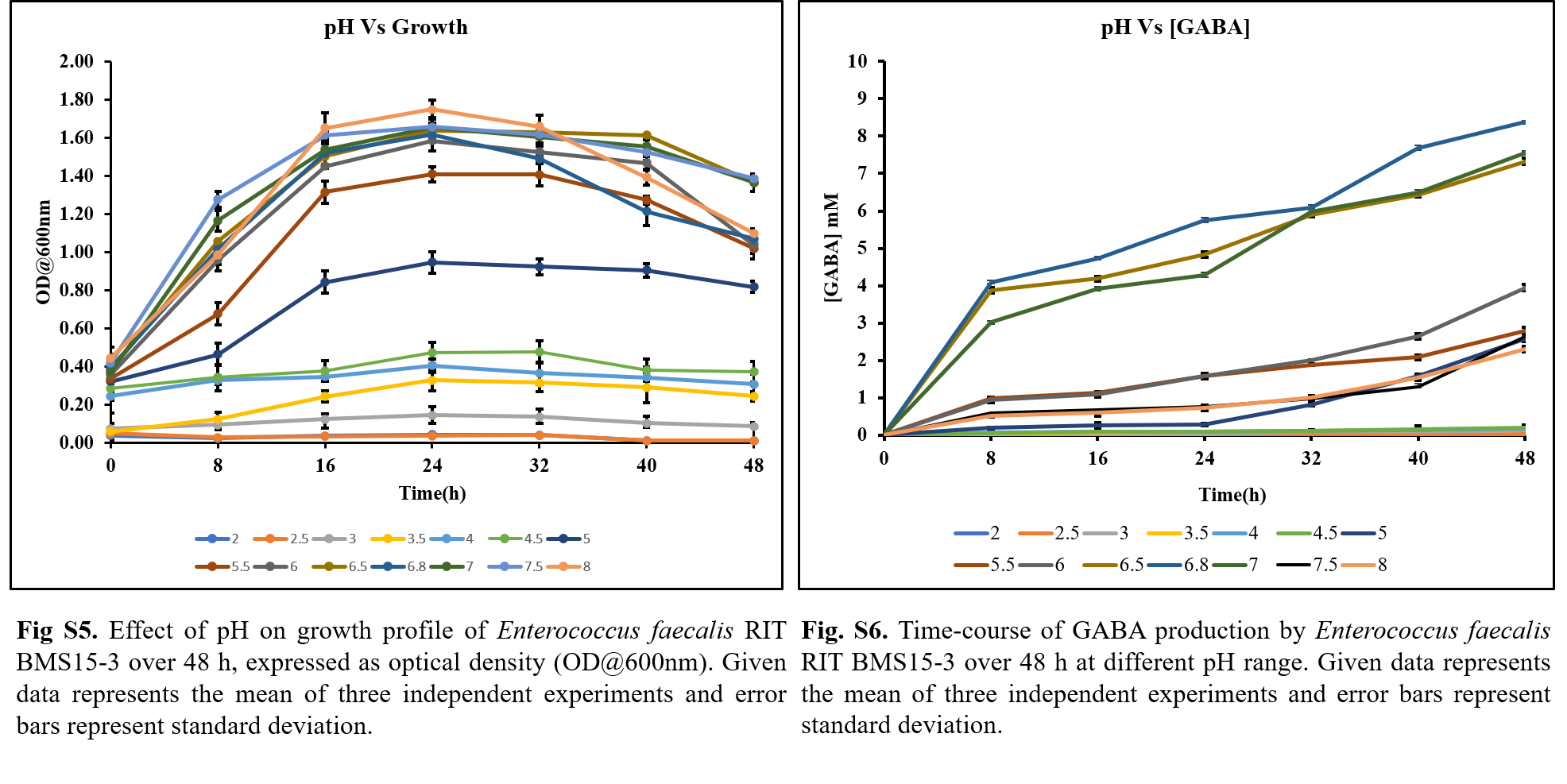

Supplement: Supplementary file 3 [file Image_3.TIF]
